# Trends and risk factors for childhood diarrhea in sub-Saharan countries (1990–2013): assessing the neighborhood inequalities

**DOI:** 10.3402/gha.v9.30166

**Published:** 2016-05-11

**Authors:** Aristide R. Bado, A. Sathiya Susuman, Eric I. Nebie

**Affiliations:** 1 Department of Statistics and Population Studies, Faculty of Natural Sciences, University of the Western Cape, Cape Town, South Africa; 2 Centre de Recherche en Santé de Nouna (CRSN), Nouna, Burkina Faso

**Keywords:** diarrheal morbidity, neighborhood inequalities, under-5 mortality, sub-Saharan Africa, Demographic and Health Survey

## Abstract

**Background:**

Diarrheal diseases are a major cause of child mortality and one of the main causes of medical consultation for children in sub-Saharan countries. This paper attempts to determine the risk factors and neighborhood inequalities of diarrheal morbidity among under-5 children in selected countries in sub-Saharan Africa over the period 1990–2013.

**Design:**

Data used come from the Demographic and Health Survey (DHS) waves conducted in Burkina Faso (1992–93, 1998–99, 2003, and 2010), Mali (1995, 2001, 2016, and 2012), Nigeria (1990, 1999, 2003, 2008, and 2013), and Niger (1992, 1998, 2006, and 2012). Bivariate analysis was performed to assess the association between the dependent variable and each of the independent variables. Multilevel logistic regression modelling was used to determine the fixed and random effects of the risk factors associated with diarrheal morbidity.

**Results:**

The findings showed that the proportion of diarrheal morbidity among under-5 children varied considerably across the cohorts of birth from 10 to 35%. There were large variations in the proportion of diarrheal morbidity across countries. The proportions of diarrheal morbidity were higher in Niger compared with Burkina Faso, Mali, and Nigeria. The risk factors of diarrheal morbidity varied from one country to another, but the main factors included the child's age, size of the child at birth, the quality of the main floor material, mother's education and her occupation, type of toilet, and place of residence. The analysis shows an increasing trend of diarrheal inequalities according to DHS rounds. In Burkina Faso, the value of the intraclass correlation coefficient (ICC) was 0.04 for 1993 DHS and 0.09 in 2010 DHS; in Mali, the ICC increased from 0.04 in 1995 to 0.16 in 2012; in Nigeria, the ICC increased from 0.13 in 1990 to 0.19 in 2013; and in Niger, the ICC increased from 0.07 in 1992 to 0.11 in 2012.

**Conclusions:**

This suggests the need to fight against diarrheal diseases on both the local and community levels across villages.

## Introduction

The morbidity and mortality related to diarrheal diseases in under-5 children are still sizeable and persistent in low-income countries, especially in sub-Saharan Africa, and pose a significant, long-standing public health concern. Diarrheal disease is one of the leading causes of morbidity and mortality in less-developed countries, especially among under-5 children (1). Fischer Walker et al. (2) estimated that in 2010, about 1,731 billion episodes of diarrhea in children younger than 5 years were recorded worldwide. Every year, nearly 760,000 under-5 children continue to die from diarrheal disease, and the majority of these deaths have been identified as being avoidable (2, 3) The burden of diarrheal morbidity lies largely in the developing world, where water and living conditions remain poor (4). In 2011, Fischer Walker et al. (2) showed that nearly three-quarters of diarrheal mortality and pneumonia mortality are concentrated in 15 high-burden countries, and among these 15 countries, 10 were from sub-Saharan Africa; namely Angola, Burkina Faso, Democratic Republic of the Congo, Ethiopia, Kenya, Mali, Niger, Nigeria, Tanzania, and Uganda. Therefore, aggressive efforts to reduce child mortality must be through the reduction or elimination of diarrheal morbidity and mortality among under-5 children, especially in low- and middle-incomes countries (5). The United Nations’ Millennium Development Goal 4, which aims to decrease the child mortality rate by two-thirds between 1990 and 2015, will not be achievable if morbidity and mortality caused through diarrhea are not curbed (6, 7).

The fight against diarrheal diseases in under-5 children was the subject of several international interventions as well as regional and national interventions in low- and middle-income countries (8, 9). In the late 1970s and early 1980s, international initiatives were implemented to reduce diarrheal mortality in under-5 children in developing countries (6). These actions included the promotion of the use of oral rehydration therapy, coupled with programs to educate caregivers on its appropriate use (10); supplementation with zinc, which has been shown to reduce the duration, severity, and complications associated with diarrhea; the promotion of hygiene; and access to drinking water in households.

Indeed, diarrheal diseases remain linked largely to living conditions, poverty, lack of hygiene, and lack of drinking water in households and in the neighborhood. The differences among cities, villages, neighborhoods, and countries with purified water supply, sanitation, drainage, and waste removal are the factors of inequalities in morbidity and mortality from diarrheal disease (11). The neighborhoods with inadequate provisions of water or sanitation and with unsanitary living conditions are often more likely to suffer the burden of diarrheal disease and record higher rates of mortality of under-5 children (11–13). The results of earlier studies have shown that lack of sanitation, availability and supply of drinking water, and lack of proper septic tanks and toilets, especially in urban suburbs, are generally expected to increase the risk of diarrheal morbidity and mortality and the infant mortality rate (14–17). Similarly, poor economic status, food scarcity, hand washing without soap, and kind of water storage were identified as risk factors for diarrheal morbidity and mortality (18–20).

Earlier studies on risk factors of morbidity and mortality caused by diarrhea have highlighted a large group of factors related to socio-economic status, living conditions for children, and factors related to etiology. Among the socio-economic variables, household poverty level; a high number of people living in the household (19) or a high number of under-5 children in the household; and maternal age, education, and working status (21) were the risk factors for morbidity and mortality from diarrheal diseases as reported in previous studies. The child's characteristics that were found to be significantly associated with the risk of diarrheal morbidity and mortality included age, gender, type of breast-feeding used, underweight, and acute malnutrition. Malnutrition and low socio-economic status are the factors that lead to the risk of mortality due to morbidity and diarrheal morbidity.

## Aim

This paper attempts to determine the risk factors and neighborhood inequalities of diarrheal morbidity among under-5 children in selected countries in sub-Saharan Africa from 1990 to 2013. Measurements of trends in diarrheal morbidity among under-5 children are important to assess the progress toward Millennium Development Goal 4, which targets the reduction of child mortality by two-thirds over the period 1990–2013.

## Methods

### Study design

This study is a comparative cross sectional. Four countries have been selected to be in this study: Burkina Faso, Mali, Nigeria, and Niger. These countries are all located in West Africa, one of the poorest parts of the world, where the indicators of morbidity and mortality of children are the most disturbing in the world. According to Fischer Walker et al. (2), these four countries were among the top 10 countries of the world that accounted for more than 52% of childhood deaths from diarrhea and pneumonia. One of the reasons that motivated the choice of these countries is that, during 1990–2013, each country has made at least the fourth rounds of Demographic and Health Survey (DHS). The availability of data on diarrheal morbidity in under-5 children, as well as information on household hygiene, type of drinking water sources used in the household, and type of sanitation used in each database from the different rounds of DHS offers an opportunity to analyze the trend of the morbidity of diarrhea in under-5 children in these countries. The data allow the research to make a comparative analysis among countries selected for this study.

### Data source

Data used come from the DHS waves conducted in Burkina Faso (1992–93, 1998–99, 2003, and 2010), Mali (1995, 2001, 2016, and 2012), Nigeria (1990, 1999, 2003, 2008, and 2013), and Niger (1992, 1998, 2006, and 2012) (Table 1). The data were obtained from the MEASURE DHS. Each of these surveys collected information from a representative sample at the national level. The DHS is based on a stratified, two-stage cluster sample. A cluster usually consists of one or a few villages in the rural area or a neighborhood in the urban environment. This type of sampling allows us to take into account the contextual effect for studying the determinants of diarrheal morbidity in the countries selected for the study.

Three types of questionnaires were administered in each survey, namely the Household, Man, and Woman questionnaires. In this paper, we used the data for birth history information of all women aged 15 to 49 interviewed for the different surveys. The birth history data set contains information on the date of birth of all the children a woman has had in her life, starting from her first child until the time of the survey. Information on child survival (dead or alive) was also collected.

### Variable specification

Diarrhea is a syndrome that can be caused by different bacterial, viral, and parasitic pathogens. In West African countries, studies show that diarrheal morbidity is caused mainly by *Escherichia coli*, *Shigella* spp., and rotavirus (22, 23).

The variables used in this study for the explanation of diarrheal morbidity in under-5 children have been selected according to the conceptual framework of risk factors for diarrhea incidence in developing countries as proposed by Genser et al. (24). This conceptual framework proposes five groups of variables considered as determinants of diarrheal morbidity. The socio-economic status of households (the standard of living in the household, the number of people in the household, age of mother, occupation, and level of education) comprises the first group of variables. The second group of variables is related to the hygiene of the immediate environment of the household. This group of variables includes the drinking water source and type of toilet used in the household. Weight at birth, breast-feeding duration, anthropometric measurements, and age and sex of the child represent the third group of potential determinants of diarrheal morbidity.

### Dependent variable

The main variable of our study is under-5 children having had diarrhea in the past 2 weeks preceding the survey. This variable was collected during each round of DHS. During the interview, to assess the prevalence of diarrheal diseases in under-5 children, mothers were asked whether their children had had diarrhea in the 2 weeks preceding the survey and if there was blood in the stool. The dependent variable then is dichotomous: yes, if the child had an episode of diarrhea in the past 2 weeks, and no, otherwise.

### Independent variables

Four groups of variables are taken into account in the analysis models. The first includes variables related to socio-economic status of the household (the number of people in household; presence of electricity; ownership of radio, television, refrigerator, bicycle, and motorcycle in the household; and the level of the mother's education and her age). The source of drinking water, type of toilet, and floor material quality are the variables related to hygiene and quality of the immediate environment of the household. Demographic information for the child includes age, weight at birth, sex, and status regarding measles vaccine.

### Data analysis

Data analysis was carried out using STATA software Version 13.1 (StataCorp. 2013. *Stata Statistical Software: Release 13*. College Station, TX: StataCorp LP). Two types of analysis were used: a bivariate analysis and a multilevel logistic regression modelling. Bivariate analysis for each independent variable was performed against the dependent variable to elicit the impact of each factor on diarrheal morbidity. The dependent variable was dichotomous, and to reflect the hierarchical structure of the data, a two-level multilevel logistic regression model, with individuals at level-1 and the local area Primary Sampling Unit (PSU) at level-2, was used to assess the effect of each variable independently on the dependent variable while controlling for the cofounders.

#### Specification of the model

In $(\frac{p_{\text{ij}}}{\text{1}-p_{\text{ij}}})=\beta_{0}+\beta_{1}x_{\text{1ij}}+\beta_{2}x_{2\text{ij}}+\ldots +\beta_{n}x_{\text{nij}}+\omega_{j}$, the random effects vector *ω*_j_ is distributed as N(0,$\sigma_{\omega }^{2}$),

where x_1_ to x_n_ represents the explanatory variables for the probability that a child aged less than 5 years i from a PSUj has suffered from diarrheal disease. β_0_ is the intercept and β(_1_ to _n_) are the fixed effect (coefficients) for the explanatory variables included in the model. *ω*_*j*_ is the PSU-level random effect.

#### Intraclass correlation and neighborhood inequalities measurement

The random effect (*ω*_*j*_) measures the variation between neighborhoods in the proportion of diarrheal morbidity among under-5 children. To quantify the neighborhood inequalities in diarrheal morbidity, we calculated the intraclass correlation (ICC), which is the percentage of the total variance among the neighborhoods. The ICC is the proportion of the total variance of diarrheal morbidity among under-5 children at the neighborhood level (PSU). The ICC quantified the inequalities or the contextual effect of diarrheal morbidity (25, 26).

### Results

**Fig. 1:**
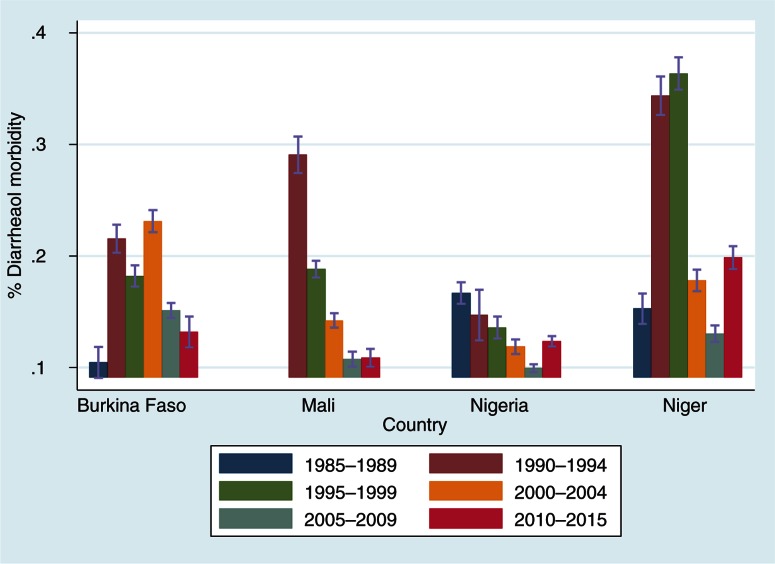
Diarrheal morbidity prevalence by cohort and country.

Figure 1 presents the prevalence of diarrheal morbidity by cohort across countries. There are variations by country and by cohort. The highest prevalence of diarrheal morbidity is among children in Niger whereas the lowest proportions were recorded among children in Nigeria.

The proportions of under-5 children who suffered from diarrheal diseases were higher in Niger (36.4%, 95% confidence interval [CI]=[34.9; 37.8]) among children of the 1995–1999 birth cohort compared with other countries. In Nigeria, the proportions of diarrheal morbidity were (14.7% [12.5; 16.9] for the 1990–1994 birth cohort; 13.6% [12.6; 14.6] for the 1995–1999 birth cohort) compared with the prevalence of other countries for the same birth cohort of children. For all countries, the results showed that diarrheal morbidity proportions among under-5 children are declining from 1995 to 2009. In Niger, although the results show an overall decrease of diarrheal morbidity, there is an increase in the last birth cohort (2010–2015) compared with the previous birth cohorts (2005–2009 and 2000–2004).

**Table 1:** Years of survey and population of under-five children by country

Country	Year of survey	1990–1995	1996–2001	2002–2008	2009–2012	2013
Burkina Faso	1992, 1998, 2003, 2010	5,096	5,071	9,361	13,716	
Mali	1995, 2001, 2006, 2012	5,231	11,055	12,388	9,582	
Nigeria	1990, 1999, 2003, 2008, 2013	6,823	3,139	5,163	25,446	28,596
Niger	1992, 1998, 2006, 2012	5,592	4,243	8,209	11,602	

Table 2 presents the results of the descriptive analysis of diarrheal morbidity. The results by countries show that the child-level variables (sex, age, size of the child at birth, and had a measles vaccine) are significantly associated with diarrheal morbidity as variables. However, there are differences among countries. For Burkina Faso, only the child-level variables are significant. For Mali, Nigeria, and Niger, the variables related to the immediate environment and hygiene (type of toilet, quality of main floor material, and source of drinking water) are associated significantly with diarrheal morbidity in under-5 children. Children from households using a traditional latrine or who do not have a toilet and those from the households with poor quality of main floor material, and using a tube well or borehole, are more likely to suffer from diarrheal disease compared with the children from households using improved toilets, having a good quality of main floor material, and who are using piped water as their main source of drinking water.

**Table 2:** Determinant variables in bivariate analysis

Country	1990–1995	1996–2001	2002–2008	2009–2012	2013
	1993	1999	2003	2010	
Burkina Faso	- Quality of the main floor*** - Sex of the child** - Child's age (months)*** - Has measles vaccine***	- Child's age (months)*** - Has measles vaccine*** - Type of toilet	- Household size*** - Has a refrigerator* - Has bicycle in the HH* - Child's age (months)*** - Has measles vaccine*** - Size of the child at birth*** - Sex of the child**	- Household size*** - Age of the mother** - Mother's educational level** - Has a refrigerator* - Has bicycle in the HH* - Child's age (months)*** - Size of the child at birth***	
Mali	1995	2001	2006	2012	
	- Age of the mother* - Place of residence*** - Mother's occupation** - Mother's education level*** - Has electricity*** - Has television*** - Type of toilet*** - Quality of main floor material*** - Sources of drinking water** - Child's age (months)*** - Size of the child at birth***	- Household size*** - Age of the mother*** - Place of residence*** - Mother's occupation*** - Has electricity*** - Has a television*** - Has a refrigerator*** - Has a bicycle*** - Has a motorcycle*** - Type of toilet*** - Quality of main floor material*** - Sources of drinking water*** - Child's age (months)*** - Has measles vaccine*** - Size of the child at birth***	- Household size** - Place of residence*** - Mother's occupation*** - Has electricity*** - Has television*** - Has a bicycle** - Has a motorcycle** - Quality of main floor material*** - Sources of drinking water*** - Child's age (months)*** - Size of the child at birth***	- Mother occupation*** - Mother's education level*** - Quality of main floor material** - Sources of drinking water*** - Sex of the child* - Child's age (months)*** - Has measles vaccine** - Size of the child at birth*	
Nigeria	1990	1999	2003	2008	2013
	- Age of the mother*** - Place of residence*** - Mother's occupation*** - Mother's education*** - Has electricity*** - Has a television*** - Has a refrigerator*** - Has a bicycle*** - Type of toilet*** - Quality of the main floor material***	- Sex of the head of the HH*** - Mother's occupation*** - Mother's education*** - Has electricity*** - Has a television*** - Has a refrigerator*** - Type of toilet*** - Quality of the main floor material*** - Child's age (months)***	- Age of the mother* - Place of residence*** - Mother's education*** - Has electricity*** - Has a television*** - Has a refrigerator*** - Has a bicycle*** - Has a motorcycle* - Type of toilet*** - Quality of the main floor material***	- Household size*** - Sex of the head of the HH*** - Age of the mother*** - Place of residence*** - Mother's education*** - Mother's education*** - Has electricity*** - Has a television*** - Has a refrigerator*** - Has a bicycle*** - Has a motorcycle***	- Household size*** - Sex of the head of the HH*** - Age of the mother*** - Place of residence*** - Mother's education*** - Mother's education*** - Has electricity*** - Has a television*** - Has a refrigerator*** - Has a bicycle*** - Type of toilet***
	- Sources of drinking water*** - Sex of the child*** - Child's age (months)*** - Has measles vaccine*** - Size of the child at birth***		- Sources of drinking water*** - Sex of the child* - Child's age (months)*** - Has measles vaccine*** - Size of the child at birth***	- Type of toilet*** - Quality of the main floor material*** - Sources of drinking water*** - Sex of the child** - Child's age (months)** - Has measles vaccine*** - Size of the child at birth***	- Quality of the main floor material*** - Sources of drinking water*** - Child's age (months)*** - Has measles vaccine*** - Size of the child at birth***
Niger	1992	1998	2006	2012	
	- Household size** - Age of the mother* - Place of residence*** - Mother's occupation*** - Mother's education level*** - Has electricity*** - Has a television*** - Has a refrigerator** - Has a motorcycle** - Type of toilet** - Quality of the main floor material*** - Sources of drinking water** - Child's age (months)*** - Has a measles vaccine*** - Size of the child at birth***	- Place of residence*** - Mother’s occupation*** - Mother’s education level*** - Has electricity*** - Has a television*** - Has a refrigerator*** - Has a motorcycle*** - Quality of the main floor material*** - Sources of drinking water*** - Child’s age (months)***	- Household size** - Age of the mother* - Place of residence*** - Mother's occupation*** - Mother's education level*** - Has electricity*** - Has a television*** - Has a refrigerator** - Has bicycle*** - Type of toilet*** - Quality of the main floor material*** - Sources of drinking water*** - Child's age (months)*** - Has a measles vaccine*** - Size of the child at birth***	- Household size*** - Age of the mother* - Place of residence*** - Mother's education level*** - Has electricity* - Has a television* - Type of toilet* - Quality of the main floor material** - Sources of drinking water*** - Child's age (months)*** - Has a measles vaccine*** - Size of the child at birth***	

* *p*<0.05

** *p*<0.01

*** *p*<0.001.

Table 3 shows that among the economic variables, presence of electricity in the household and ownership of a television and refrigerator are associated with diarrheal morbidity in under-5 children in Mali, Niger, and Nigeria. The children living in rural areas and those of mothers who have not been to school are more at risk for diarrheal disease in Mali, Nigeria, and Niger compared with children living in urban areas and whose mothers are educated. The occupation of the mother also is associated significantly with diarrheal disease in under-5 children in Mali, Nigeria, and Niger.

**Table 3:** Significant Variables from the Multilevel Logistic Regression of with diarrheal morbidity among children under-five years old in Burkina, Mali, Nigeria and Niger (cf. Supplementary file for full table)

Country	1990–1995	1996–2001	2002–2008	2009–2012	2013
Burkina Faso	1993	1999	2003	2010	
	- Quality of the main floor material** - Sex of the child** - Child's age (months)*** - Size of the child at birth**	- Mother's occupation** - Child's age (months)***	- Household size** - Has a bicycle* - Sex of the child** - Child's age (months)*** - Has a measles vaccine* - Size of the child at birth**	- Mother's occupation** - Type of toilet* - Child's age (months)*** - Size of the child at birth***	
Mali	1995	2001	2006	2012	
	- Mother's occupation** - Mother's education level* - Quality of the main floor material*** - Child's age (months)*** - Size of the child at birth*	- Household size* - Place of residence* - Mother's occupation** - Has a bicycle** - Type of toilet** - Sources of drinking water** - Child's age (months)***	- Place of residence** - Mother's occupation* - Child's age (months)*** - Has a measles vaccine* - Size of the child at birth**	- Mother's occupation*** - Type of toilet* - Child's age (months)***	
Nigeria	1990	1999	2003	2008	2013
	- Place of residence** - Mother's occupation** - Mother's education level* - Has electricity* - Has a refrigerator* - Type of toilet** - Sex of the child** - Child's age (months)*** - Size of the child at birth*	- Mother's occupation* - Mother's education level* - Child's age (months)***	- Mother's occupation* - Mother's education level* - Quality of the main floor material** - Sex of the child* - Child's age (months)*** - Size of the child at birth**	- Mother's education level* - Has electricity* - Has a television* - Type of toilet** - Quality of the main floor material** - Sources of drinking water** - Sex of the child*** - Child's age (months)*** - Size of the child at birth**	- Type of toilet** - Child's age (months)*** - Size of the child at birth**
Niger	1992	1998	2006	2012	
	- Sex of the head of the HH* - Place of residence* - Mother's occupation** - Mother's education level** - Child's age (months)*** - Size of the child at birth**	- Mother's occupation** - Quality of the main floor material* - Child's age (months)***	- Mother's occupation** - Mother's education level* - Child's age (months)*** - Size of the child at birth*	- Household size* - Mother's occupation** - Quality of the main floor material** - Child's age (months)*** - Size of the child at birth*	

* *p*<0.05

** *p*<0.01

*** *p*<0.001.

In all four countries, age of the child is associated significantly with diarrheal disease among under-5 children. The risk of diarrheal morbidity among under-5 children has a U shape according to the child's age (Supplementary file). Compared with children of 0 to 6 months, the odds ratio is between 1.5 and 3.7 for children whose age is between 7 and 36 months, whereas after 37 months, the risk of diarrheal disease is reduced (OR<1) (Supplementary file).

The results of the four DHSs conducted in Burkina Faso (Table 3) show that variables related to the child level are those most significantly associated with diarrheal morbidity in under-5 children. Male children, those who had a low birth weight, and those aged 7 to 36 months are more likely to have a diarrheal morbidity compared with female children and those who had a normal birth weight. The quality of main floor material, the type of toilet, the mother's occupation, had measles vaccine, and household sizes were associated significantly with diarrheal morbidity for the Burkina DHS. The child's age and occupation of the mother are the two significant variables for the four rounds of DHS in Mali. Children of mothers who trade or have agriculture as their main activity are more likely to suffer from diarrheal morbidity compared with children of mothers without a primary occupation. Low weight at birth and rural residence are likely to increase the likelihood of a diarrheal disease in under-5 children in Mali. Children living in rural areas were 1.3 (2001 DHS) and 1.5 (2006 DHS) more likely to have a diarrheal disease compared with children living in urban areas (Supplementary file).

The effect (Table 3) of variables, namely quality of the main floor material, the types of toilet, and the source of drinking water in Mali is significant for the explanation of diarrheal morbidity in under-5 children. Children in households using traditional toilets and those of households without toilets are at higher risk for diarrheal disease compared with children from households using a flush toilet or upgraded latrine. Regarding Nigeria, the variables related to the child (age, sex, and weight at birth) are factors associated with diarrheal morbidity in under-5 children. The results also find that variables related to the mother (occupation and education level) and socio-economic variables (electricity in the household and the possession of goods such as refrigerators and televisions) are associated with diarrheal morbidity.

**Table 4:** Random Effects of the Multilevel Logistic Regression of diarrheal morbidity among children under-five years old in Burkina, Mali, Nigeria and Niger

Country	Random Effects	1990–1995	1996–2001	2002–2008	2009–2012	2013
Burkina Faso	Community Level SD	0.39 (0.29; 0.54)	0.46 (0.36; 0.58)	0.50 (0.43; 0.59)	0.56 (0.49; 0.65)	
	ICC	0.04 (0.2; 0.08)	0.06 (0.04; 0.09)	0.07 (0.05; 0.10)	0.09 (0.07; 0.11)	
Mali	Community Level SD	0.37 (0.28; 0.50)	0.50 (0.43; 0.59)	0.70 (0.60; 0.80)	0.80 (0.70; 0.93)	
	ICC	0.04 (0.02; 0.07)	0.07 (0.05; 0.10)	0.13 (0.10; 0.16)	0.16 (0.12; 0.21)	
Nigeria	Community Level SD	0.71 (0.60; 0.84)	0.63 (0.47; 0.84)	0.77 (0.6; 0.9)	0.82 (0.75; 0.91)	0.87 (0.80; 0.95)
	ICC	0.13 (0.10; 0.17)	0.11 (0.06; 0.18)	0.15 (0.11; 0.21)	0.17 (0.14; 0.20)	0.19 (0.16; 0.21)
Niger	Community Level SD	0.50 (0.40; 0.60)	0.43 (0.35; 0.56)	0.60 (0.51; 0.70)	0.65 (0.56; 0.75)	
	ICC	0.07 (0.05; 0.10)	0.06 (0.04; 0.09)	0.10 (0.07; 0.13)	0.11 (0.09; 0.15)	

ICC=intraclass correlation coefficient.

Age of the child and birth weight are significantly associated with diarrheal disease in under-5 children in Niger. Children who had a low birth weight are between 1.2 and 1.4 times more likely to have a diarrheal disease compared with children who had a normal birth weight (Supplementary file).

**Fig. 2:**
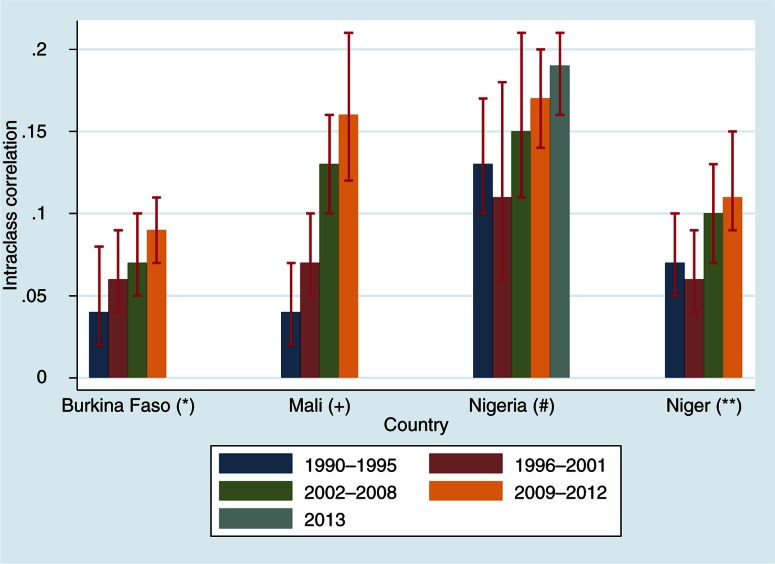
Trend of intra-class correlation by country. *=Burkina DHS 1993, 1999, 2003, 2010; +=Mali DHS 1995, 2001, 2006, 2012; #=Nigeria DHS 1990, 1999, 2003, 2008, 2013; **=Niger DHS 1992, 1998, 2006, 2012.

Table 4 and Fig. 2 present the random effect estimates of the multilevel regression analysis of diarrheal morbidity of each country. The ICC value allows us to analyze the neighborhood disparities in diarrheal morbidity. As shown in Fig. 2, Nigeria (0.19) and Mali (0.16) are the countries where we observe large neighborhood disparities in diarrheal morbidity in under-5 children. Low diarrheal morbidity inequalities are recorded in Burkina Faso (0.09). The analysis shows an increasing trend of diarrheal inequalities according to DHS rounds. In Burkina Faso, the value of the ICC was 0.04 for the 1993 DHS, while it was 0.09 in 2010 DHS; in Mali, the ICC increased from 0.04 in 1995 to 0.16 in 2012; in Nigeria, the ICC increased from 0.13 in 1990 to 0.19 in 2013; and in Niger, the ICC increased from 0.07 in 1992 to 0.11 in 2012 (Supplementary file). These results show that the neighborhood disparities of diarrheal morbidity among under-5 children have increased in all four countries.

## Discussion

### Main findings

The findings showed that the proportion of diarrheal morbidity among under-5 children varied considerably across the cohorts of birth from 10 to 35%, and we also observed large variations in the proportions of diarrheal morbidity across countries. The proportions of diarrheal morbidity are higher in Niger compared with Burkina, Mali, and Nigeria, where lower proportions were reported. Studies showed that mortality caused by diarrheal diseases among under-5 children in low- and middle-income countries has declined about 30% over the past two decades (2).

Our results showed that risk factors of diarrheal morbidity varied from one country to another, but the main factors included child's age, size of the child at birth, the quality of the main floor material, mother's education and her occupation, type of toilet, and place of residence. We observed high neighborhood disparities in diarrheal morbidity in Nigeria and Mali and low neighborhood disparities in Niger and Burkina Faso.

### Strengths and limitations

Previous studies on risk factors for diarrheal morbidity and mortality in low- and middle-income countries often were interested in individual factors. This study used data from all DHS rounds conducted in Burkina Faso, Mali, Nigeria, and Niger during the 1990–2013 period, corresponding to cohorts of children born between 1986 and 2013. The use of these rounds of DHS data allowed the researchers first to study the trend of the prevalence of diarrheal morbidity among under-5 children and, second, to make a comparative analysis across countries. One of the strengths of this study is to have investigated and quantified the magnitude of neighborhood inequalities in diarrheal morbidity in the selected countries of our study. Diarrheal morbidity in the DHS was captured by asking mothers if their children had had diarrhea in the 2 weeks preceding the survey and, if so, whether there was blood in the stool. This methodology, based on the mother's statement, contained deficiencies related to problems of recall (2 weeks) on the one hand and, on the other, the problems associated with the uncertainty of the mother's statement on the state of her child's health.

### Interpretation of the results

Previous studies have shown that during the past three decades, thanks to the introduction of oral rehydration solutions and zinc supplementation in the treatment of diarrhea, the prevalence of diarrheal diseases has been reduced drastically in low-income countries (6, 27). Santosham et al. (6) showed that with the distribution of oral solutions, the number of child deaths caused by diarrhea dropped from 4.6 million in 1980 to 3.3 million in 1990 and to 1.3 million in 2008. These significant declines recorded in the different countries would be the result of action undertaken since the late 1970s to fight against diarrheal diseases through the introduction of drugs and, especially, through awareness and promotion of hygiene. Important activities have been undertaken including training of health workers, participation of religious leaders, educational campaigns in schools, and modifications to the use of oral rehydration formulations to fit local traditions and beliefs. Mass media campaigns have been set up around the world, and many political leaders and celebrities have endorsed the use of oral rehydration solution (6). In the Niger, we found an increased proportion of diarrheal morbidity among the children born after 2010. This result was not expected given the politic against diarrheal diseases through the distribution of oral rehydration salts, with low-osmolarity salts introduced in 2006 in Niger (28).

We found that child's age is a risk factor of diarrheal morbidity, and its effect is significant for all countries and for each of the rounds of DHS. The results showed that children aged 7 to 36 months are more likely to experience diarrheal disease than children aged to 6 months, and after 36 months, the risk of suffering from a diarrheal disease becomes low. Similar results were found in Ethiopia (29), Iraq (30), Egypt (31), Turkey (32), and India (16, 33). It seems that children aged 6 to 36 months have an immune system not yet sufficiently developed to protect against the diseases compared with children aged less than 6 months who benefit directly from their mother's immune system (30). We also found that children with a small size (low weight) at birth are more likely to have been reported as having been associated with diarrheal morbidity than children who had a large size at birth. Results of earlier studies have shown that malnutrition and underweight in children are risk factors in diarrheal morbidity (34–37).

According to the results of the present study, in Burkina Faso (1993 and 2003 DHS) and in Nigeria (1990, 2003, and 2003 DHS), male children are more likely to have been reported as being associated with diarrheal morbidity than female children. Similar results were found in previous studies (20, 30, 32, 33, 38, 39). This result is not surprising as the results of both morbidity and mortality studies of under-5 children in sub-Saharan countries often have found an excess mortality in male children in early life (40–42). Studies carried out in developing countries reveal that mother's education level is an important factor of diarrheal morbidity and death among under-5 children, and children from less-educated mothers are more likely to experience diarrheal diseases than children of more-educated mothers (30, 43, 44).

Maternal education and her occupation are factors that provide information about the level of autonomy of the woman who could empower herself to take care of her child. The effect of the quality of the immediate environment as a risk factor in diarrheal morbidity in under-5 children was revealed as significant in previous studies (29, 38, 39, 45, 46). We found that the poor quality of main floor material (in Burkina, Mali, Niger, and Nigeria), the type of latrine used (in Mali and Nigeria), and the source of drinking water (in Mali and Nigeria) are associated significantly with the risk of diarrheal morbidity.

Our study found high neighborhood inequalities in diarrheal morbidity in the selected countries, while the results showed a decline in the overall prevalence of diarrhea morbidity among under-5 children. These neighborhood inequalities show that the decline in prevalence of diarrheal morbidity hides huge disparities among neighborhoods and villages in the countries under study. These results inform us that in some villages or neighborhoods in big cities, the prevalence of diarrheal diseases has lowered greatly, while for some neighborhoods or villages, the proportions of diarrheal morbidity are still high. Neighborhood inequalities observed may be explained in part by the quality of the immediate environment and the poor quality of the drinking water experienced in some villages and suburbs around large cities.

## Conclusion

In this study, we identified a decrease in the proportions of diarrheal morbidity among under-5 children in Burkina Faso, Mali, Nigeria, and Niger during the period between 1990 and 2013. However we have observed a growing trend of inequalities among neighborhoods and villages of countries selected for the study. This result shows that the decline in the prevalence of diarrheal morbidity is not uniform in the different villages and neighborhoods, and in some places, under-5 children still suffer from the burden of diarrheal disease. This suggests the need to fight against diarrheal diseases on both the local and community level across villages and countries. Successful implementation of an integrated plan requires the commitment of families, health care providers, and key actors in water and sanitation.

## Supplementary Material

Trends and risk factors for childhood diarrhea in sub-Saharan countries (1990–2013): assessing the neighborhood inequalities

## Paper context

Health inequalities of children in Sub-Saharan Africa
