# Supplementary material for: Trends and risk factors for childhood diarrhea in sub-Saharan countries (1990–2013): assessing the neighborhood inequalities
Source: Glob Health Action. 2016 May 11;9:10.3402/gha.v9.30166. doi: 10.3402/gha.v9.30166 (PMC4865764; doi:10.3402/gha.v9.30166)
Supplement: Trends and risk factors for childhood diarrhea in sub-Saharan countries (1990–2013): assessing the neighborhood inequalities [file GHA-9-30166-s001.doc]

Table 5: Determinant Variables in Bivariate Analysis

|  | **Burkina Faso** | | | | | | | |
| --- | --- | --- | --- | --- | --- | --- | --- | --- |
| ***Variable*** | **1993** | | **1998** | | **2003** | | **2010** | |
|  | **No** | **Yes** | **No** | **Yes** | **No** | **Yes** | **No** | **Yes** |
| ***n*** | ***4089*** | ***1010*** | ***4,056*** | ***1,015*** | ***7,485*** | ***1,876*** | ***11,685*** | ***2,031*** |
| **Household size** | **ns** | | **ns** | | ******* | | ******* | |
| 1-3 | 78.7 | 21.3 | 78.7 | 21.3 | 72.6 | 27.4 | 81.3 | 18.7 |
| 4-6 | 79.3 | 20.7 | 81.6 | 18.4 | 80.8 | 19.2 | 85.6 | 14.4 |
| 7&+ | 80.7 | 19.3 | 79.4 | 20.6 | 80.3 | 19.7 | 85.5 | 14.5 |
| **Sex of the head of household** | **ns** | | **ns** | | **ns** | | **ns** | |
| Male | 80.0 | 20.0 | 80.0 | 20.0 | 80.11 | 19.89 | 85.3 | 14.8 |
| Female | 83.8 | 16.3 | 80.5 | 19.5 | 76.82 | 23.18 | 84.4 | 15.6 |
| **Age of the mother** | **ns** | | **ns** | | **ns** | | ****** | |
| 15-19 | 76.2 | 23.8 | 76.9 | 23.1 | 76.5 | 23.5 | 80.0 | 20.0 |
| 20-24 | 79.5 | 20.6 | 78.0 | 22.1 | 78.6 | 21.4 | 84.2 | 15.8 |
| 25-29 | 80.4 | 19.6 | 80.6 | 19.4 | 81.0 | 19.0 | 85.6 | 14.4 |
| 30-34 | 81.1 | 18.9 | 81.1 | 18.9 | 80.5 | 19.5 | 85.9 | 14.1 |
| 35-39 | 81.8 | 18.2 | 80.6 | 19.4 | 80.3 | 19.7 | 86.0 | 14.0 |
| 40-44 | 79.6 | 20.4 | 82.8 | 17.2 | 81.2 | 18.8 | 85.6 | 14.4 |
| 45-49 | 78.2 | 21.8 | 78.1 | 22.0 | 78.9 | 21.1 | 88.5 | 11.5 |
| **Place of residence** | **ns** | | **ns** | | **ns** | | **ns** | |
| Urban | 81.4 | 18.6 | 80.3 | 19.7 | 80.2 | 19.8 | 84.7 | 15.3 |
| Rural | 79.6 | 20.5 | 79.9 | 20.1 | 79.9 | 20.1 | 85.3 | 14.7 |
| **Mother Occupation** | **ns** | | **ns** | | **ns** | | **ns** | |
| Not working | 81.5 | 18.5 | 82.6 | 17.4 | 77.8 | 22.2 | 86.4 | 13.6 |
| Sale | 78.9 | 21.1 | 79.7 | 20.3 | 79.7 | 20.3 | 84.9 | 15.1 |
| Agriculter | 78.5 | 21.5 | 78.8 | 21.2 | 80.4 | 19.6 | 85.3 | 14.8 |
| Manual | 81.4 | 18.6 | 80.6 | 19.4 | 77.9 | 22.1 | 83.4 | 16.6 |
| **Mother's education level** | **ns** | | **ns** | | **ns** | | ****** | |
| No Education | 80.0 | 20.0 | 80.1 | 19.9 | 80.0 | 20.0 | 85.6 | 14.4 |
| 1-6 years | 79.3 | 20.7 | 78.5 | 21.5 | 78.1 | 22.0 | 82.6 | 17.4 |
| 7&+ years | 85.2 | 14.8 | 80.9 | 19.1 | 83.1 | 16.9 | 84.4 | 15.6 |
| **Has Electricity** | **ns** | | **ns** | | **ns** | | **ns** | |
| No | 79.8 | 20.2 | 79.8 | 20.3 | 79.8 | 20.2 | 85.2 | 14.8 |
| Yes | 83.4 | 16.6 | 83.5 | 16.5 | 82.4 | 17.6 | 84.8 | 15.2 |
| **Has Television** | **ns** | | **ns** | | **ns** | | **ns** | |
| No | 79.9 | 20.1 | 79.9 | 20.1 | 79.7 | 20.3 | 85.2 | 14.8 |
| Yes | 83.4 | 16.6 | 81.6 | 18.4 | 82.3 | 17.7 | 85.3 | 14.7 |
| **Has Refrigerator** | **ns** | | **ns** | | ***** | | ***** | |
| No | 80.0 | 20.0 | 80.0 | 20.0 | 79.8 | 20.2 | 85.1 | 14.9 |
| Yes | 85.1 | 15.0 | 79.0 | 21.1 | 85.2 | 14.8 | 87.6 | 12.4 |
| **Has Bicycle** | **ns** | | **ns** | | ***** | | ***** | |
| No | 80.1 | 19.9 | 77.9 | 22.1 | 77.6 | 22.4 | 83.0 | 17.0 |
| Yes | 80.2 | 19.8 | 80.4 | 19.6 | 80.4 | 19.6 | 85.5 | 14.6 |
| **Has Motorcycle** | **ns** | | **ns** | | **ns** | | **ns** | |
| No | 79.3 | 20.7 | 79.9 | 20.1 | 79.5 | 20.5 | 84.9 | 15.1 |
| Yes | 81.6 | 18.5 | 80.1 | 19.9 | 81.2 | 18.8 | 85.7 | 14.3 |
| ***Immediat environment and hygiene*** |  |  |  |  |  |  |  |  |
| **Type of toilette** | **ns** | | **ns** | | **ns** | | **ns** | |
| Flush/upgraded latrin | 85.7 | 14.3 | 72.4 | 27.6 | 72.4 | 27.6 | 78.9 | 21.1 |
| Traditional latrines | 80.7 | 19.3 | 78.8 | 21.2 | 79.4 | 20.6 | 84.9 | 15.1 |
| Lack of toilet | 79.8 | 20.2 | 80.7 | 19.4 | 80.2 | 19.8 | 85.5 | 14.6 |
| Other type | 78.3 | 21.7 | 74.7 | 25.3 | 81.4 | 18.6 | 85.1 | 14.9 |
| **Quality of Main floor material** | ******* | | **ns** | | **ns** | | **ns** | |
| Poor | 78.6 | 21.5 | 79.9 | 20.2 | 79.5 | 20.5 | 85.8 | 14.2 |
| Middle | 82.6 | 17.4 | 80.5 | 19.5 | 80.8 | 19.2 | 84.5 | 15.5 |
| Rich | 82.6 | 17.4 | 75.0 | 25.0 | 79.4 | 20.6 | 83.2 | 16.8 |
| **Sources of drinking water** | **ns** | | **ns** | | **ns** | | **ns** | |
| Piped/Public tap/stan | 80.5 | 19.5 | 79.1 | 20.9 | 79.7 | 20.3 | 84.4 | 15.6 |
| Tube well or borehole | 80.0 | 20.0 | 80.3 | 19.7 | 80.5 | 19.5 | 85.6 | 14.4 |
| Well | 83.5 | 16.5 | 79.6 | 20.4 | 79.7 | 20.3 | 85.1 | 14.9 |
| Spring/river/lake/other | 78.7 | 21.3 | 77.2 | 22.8 | 80.0 | 20.0 | 85.0 | 15.1 |
| ***Child level variables*** |  |  |  |  |  |  |  |  |
| Sex of the child | ** | | ns | | *** | | ns | |
| Female | 81.75 | 18.25 | 80.3 | 19.7 | 81.3 | 18.7 | 85.4 | 14.6 |
| Male | 78.65 | 21.35 | 79.7 | 20.3 | 78.7 | 21.3 | 85.0 | 15.0 |
| **Age in months** | ******* | | ******* | | ******* | | ******* | |
| 0-6 | 82.07 | 17.93 | 85.1 | 14.9 | 84.3 | 15.8 | 89.1 | 10.9 |
| 07-12 | 71.22 | 28.78 | 67.8 | 32.2 | 68.8 | 31.2 | 76.4 | 23.6 |
| 13-18 | 64.49 | 35.51 | 68.4 | 31.6 | 68.2 | 31.8 | 73.4 | 26.7 |
| 19-24 | 74.64 | 25.36 | 71.4 | 28.6 | 71.2 | 28.8 | 78.7 | 21.3 |
| 25-30 | 74.79 | 25.21 | 75.0 | 25.1 | 73.4 | 26.6 | 80.7 | 19.3 |
| 31-36 | 78.91 | 21.09 | 77.5 | 22.5 | 81.6 | 18.4 | 85.5 | 14.6 |
| 37-42 | 85.79 | 14.21 | 83.6 | 16.4 | 84.4 | 15.6 | 90.1 | 9.9 |
| 42-48 | 86.78 | 13.22 | 87.4 | 12.7 | 87.4 | 12.6 | 92.4 | 7.6 |
| 48-59 | 93.62 | 6.38 | 92.4 | 7.6 | 91.4 | 8.7 | 94.6 | 5.4 |
| **Has Measles vaccine** | ******* | | ******* | | ******* | | **ns** | |
| **No** | 77.42 | 22.58 | 78.4 | 21.6 | 78.1 | 21.9 | 85.4 | 14.7 |
| **Yes** | 82.12 | 17.88 | 81.7 | 18.3 | 81.7 | 18.4 | 85.1 | 14.9 |
| **Size of child at birth** | **ns** | | **ns** | | ******* | | ******* | |
| Large | 80.52 | 19.48 | 78.7 | 21.3 | 78.3 | 21.8 | 83.2 | 16.8 |
| Average | 81.2 | 18.8 | 81.5 | 18.5 | 81.3 | 18.7 | 86.7 | 13.3 |
| Small | 77.19 | 22.81 | 78.6 | 21.4 | 77.8 | 22.2 | 83.1 | 16.9 |
| DK (Don't know) | 81.75 | 18.25 | 92.9 | 7.1 | 89.3 | 10.7 | 93.8 | 6.3 |

**=p-value<0.05; **=p-value <0.01; ***=p-value <0.001, ns= no significant*

Appendix 1: Determinant Variables in Bivariate Analysis (Count…)

|  | **Mali** | | | | | | | |
| --- | --- | --- | --- | --- | --- | --- | --- | --- |
| ***Variable*** | **1995** | | **2001** | | **2006** | | **2012** | |
|  | **No** | **Yes** | **No** | **Yes** | **No** | **Yes** | **No** | **Yes** |
| ***n*** | ***3,857*** | ***1,374*** | ***8,872 2,183*** |  | ***10,938*** | ***1,450*** | ***8,738*** | ***844*** |
| **Household size** | **ns** | | ******* | | ****** | | **ns** | |
| 1-3 | 76.0 | 24.1 | 75.3 | 24.7 | 85.6 | 14.4 | 90.1 | 9.9 |
| 4-6 | 72.7 | 27.3 | 80.3 | 19.7 | 88.9 | 11.1 | 91.2 | 8.8 |
| 7&+ | 74.1 | 26.0 | 81.2 | 18.8 | 88.3 | 11.7 | 91.3 | 8.7 |
| **Sex of the head of household** | **ns** | | **ns** | | **ns** | | **ns** | |
| Male | 73.8 | 26.2 | 80.2 | 19.8 | 88.5 | 11.5 | 91.1 | 8.9 |
| Female | 73.1 | 26.9 | 81.3 | 18.7 | 86.3 | 13.7 | 92.3 | 7.7 |
| **Age of the mother** | ***** | | ******* | | **ns** | | **ns** | |
| 15-19 | 76.7 | 23.3 | 76.7 | 23.3 | 86.4 | 13.6 | 89.1 | 10.9 |
| 20-24 | 75.0 | 25.0 | 79.9 | 20.1 | 87.9 | 12.1 | 90.9 | 9.1 |
| 25-29 | 75.4 | 24.7 | 81.6 | 18.4 | 88.3 | 11.7 | 91.6 | 8.4 |
| 30-34 | 71.9 | 28.2 | 79.5 | 20.5 | 88.7 | 11.3 | 91.9 | 8.1 |
| 35-39 | 72.2 | 27.8 | 81.2 | 18.8 | 88.8 | 11.2 | 90.4 | 9.6 |
| 40-44 | 68.9 | 31.1 | 79.4 | 20.6 | 89.7 | 10.3 | 91.4 | 8.6 |
| 45-49 | 64.7 | 35.3 | 84.7 | 15.4 | 88.8 | 11.2 | 94.4 | 5.6 |
| **Place of residence** | ****** | | ******* | | ******* | | **ns** | |
| Urban | 76.3 | 23.7 | 85.3 | 14.8 | 91.5 | 8.5 | 90.3 | 9.7 |
| Rural | 72.6 | 27.4 | 78.9 | 21.1 | 86.9 | 13.1 | 91.5 | 8.5 |
| **Mother Occupation** | ****** | | ******* | | ******* | | ******* | |
| Not working | 76.1 | 23.9 | 80.6 | 19.4 | 90.8 | 9.24 | 94.0 | 6.0 |
| Sale | 72.2 | 27.8 | 82.5 | 17.5 | 88.3 | 11.66 | 87.8 | 12.2 |
| Agriculter | 70.4 | 29.6 | 79.4 | 20.6 | - | - | 87.6 | 12.4 |
| Manual | 74.4 | 25.6 | 76.8 | 23.3 | 86.0 | 14.04 | 89.2 | 10.9 |
| **Mother's education level** | ******* | | **ns** | | **ns** | | ******* | |
| No Education | 72.4 | 27.6 | 79.9 | 20.1 | 88.1 | 12.0 | 91.7 | 8.3 |
| 1-6 years | 79.5 | 20.5 | 81.6 | 18.4 | 88.9 | 11.1 | 89.2 | 10.8 |
| 7&+ years | 85.0 | 15.0 | 83.7 | 16.3 | 90.9 | 9.1 | 88.4 | 11.6 |
| **Has Electricity** | ******* | | ******* | | ******* | | **ns** | |
| No | 73.1 | 26.9 | 79.6 | 20.4 | 87.5 | 12.5 | 91.5 | 8.5 |
| Yes | 82.6 | 17.4 | 86.1 | 14.0 | 92.5 | 7.5 | 90.4 | 9.6 |
| **Has Television** | ******* | | ******* | | ******* | | **ns** | |
| No | 72.7 | 27.3 | 79.3 | 20.7 | 87.3 | 12.7 | 91.4 | 8.6 |
| Yes | 83.8 | 16.2 | 85.8 | 14.2 | 91.4 | 8.7 | 90.7 | 9.3 |
| **Has Refrigerator** | ******* | | ******* | | ***** | | **ns** | |
| No | 73.4 | 26.7 | 79.9 | 20.1 | 88.2 | 11.8 | 91.3 | 8.7 |
| Yes | 85.2 | 14.8 | 86.5 | 13.5 | 91.8 | 8.2 | 89.5 | 10.5 |
| **Has Bicycle** | **ns** | | ******* | | ****** | | **ns** | |
| No | 73.3 | 26.7 | 78.0 | 22.0 | 89.1 | 10.9 | 91.2 | 8.8 |
| Yes | 74.3 | 25.7 | 82.3 | 17.7 | 87.5 | 12.5 | 91.2 | 8.8 |
| **Has Motorcycle** | **ns** | | ******* | | ****** | | **ns** | |
| No | 73.2 | 26.8 | 79.5 | 20.5 | 87.7 | 12.3 | 91.2 | 8.8 |
| Yes | 75.7 | 24.3 | 82.7 | 17.3 | 89.3 | 10.7 | 91.2 | 8.8 |
| ***Immediat environment and hygiene*** |  |  |  |  |  |  |  |  |
| **Type of toilette** | ******* | | ******* | | **ns** | | **ns** | |
| Flush/upgraded latrin | 88.6 | 11.4 | 86.6 | 13.4 | 92.1 | 7.9 | 92.5 | 7.6 |
| Traditional latrines | 75.2 | 24.8 | 81.3 | 18.7 | 88.3 | 11.7 | 91.2 | 8.8 |
| Lack of toilet | 69.2 | 30.8 | 75.3 | 24.7 | 88.0 | 12.0 | 90.2 | 9.8 |
| Other type | 84.9 | 15.2 | 81.5 | 18.5 | 89.2 | 10.8 | 90.1 | 9.9 |
| **Quality of Main floor material** | ******* | | ******* | | ******* | | ****** | |
| Poor | 71.6 | 28.4 | 79.2 | 20.9 | 87.7 | 12.3 | 91.7 | 8.3 |
| Middle | 81.9 | 18.1 | 85.6 | 14.4 | 90.1 | 9.9 | 90.0 | 10.0 |
| Rich | 82.0 | 18.0 | 82.5 | 17.5 | 91.6 | 8.4 | 88.8 | 11.2 |
| **Sources of drinking water** | ****** | | ******* | | ******* | | ******* | |
| Piped/Public tap/stan | 75.1 | 25.0 | 84.6 | 15.4 | 91.1 | 8.9 | 89.4 | 10.6 |
| Tube well or borehole | 73.8 | 26.2 | 79.2 | 20.8 | - | - | 92.3 | 7.7 |
| Well | 63.1 | 36.9 | 80.4 | 19.6 | 87.5 | 12.6 | 91.6 | 8.4 |
| Spring/river/lake/other | 81.8 | 18.2 | 73.6 | 26.4 | 87.5 | 12.5 | 93.9 | 6.2 |
| ***Child level variables*** |  |  |  |  |  |  |  |  |
| **Sex of the child** | **ns** | | **ns** | | **ns** | | ***** | |
| Female | 74.6 | 25.4 | 81.0 | 19.1 | 88.7 | 11.3 | 91.8 | 8.2 |
| Male | 72.9 | 27.1 | 79.6 | 20.4 | 87.9 | 12.1 | 90.6 | 9.4 |
| **Age in months** | ******* | | ******* | | ******* | | ******* | |
| 0-6 | 84.5 | 15.6 | 82.8 | 17.2 | 93.2 | 6.8 | 92.5 | 7.5 |
| 07-12 | 68.5 | 31.5 | 69.0 | 31.0 | 79.9 | 20.1 | 85.8 | 14.2 |
| 13-18 | 65.9 | 34.1 | 71.5 | 28.5 | 80.4 | 19.6 | 84.7 | 15.3 |
| 19-24 | 69.7 | 30.3 | 73.4 | 26.6 | 81.0 | 19.1 | 88.8 | 11.2 |
| 25-30 | 74.0 | 26.0 | 77.5 | 22.6 | 86.8 | 13.2 | 90.1 | 9.9 |
| 31-36 | 75.0 | 25.0 | 80.0 | 20.0 | 89.4 | 10.6 | 92.5 | 7.5 |
| 37-42 | - | - | 85.6 | 14.4 | 90.3 | 9.8 | 93.3 | 6.7 |
| 42-48 | - | - | 86.1 | 13.9 | 94.7 | 5.3 | 94.4 | 5.6 |
| 48-59 | - | - | 90.1 | 10.0 | 95.3 | 4.7 | 94.8 | 5.2 |
| **Has Measles vaccine** | **ns** | | ******* | | **ns** | | ****** | |
| No | 74.5 | 25.5 | 78.1 | 21.9 | 87.7 | 12.3 | 92.3 | 7.8 |
| Yes | 72.5 | 27.5 | 83.3 | 16.7 | 88.8 | 11.2 | 90.5 | 9.5 |
| **Size of child at birth** | ******* | | ******* | | ******* | | ***** | |
| Large | 73.2 | 26.8 | 80.9 | 19.1 | 88.8 | 11.2 | 90.7 | 9.3 |
| Average | 75.2 | 24.8 | 81.5 | 18.5 | 88.9 | 11.1 | 91.0 | 9.1 |
| Small | 69.9 | 30.1 | 76.8 | 23.2 | 85.4 | 14.6 | 92.5 | 7.6 |
| DK (Don't know) | 87.7 | 12.3 | 82.3 | 17.7 | 93.3 | 6.7 | 94.4 | 5.6 |

**=p-value<0.05; **=p-value <0.01; ***=p-value <0.001, ns= no significant*

Appendix 1: Determinant Variables in Bivariate Analysis (Count…)

|  | **Nigeria** | | | | | | | | | |
| --- | --- | --- | --- | --- | --- | --- | --- | --- | --- | --- |
| ***Variable*** | **1990** | | **1999** | | **2003** | | **2008** | | **2013** | |
|  | **No** | **Yes** | **No** | **Yes** | **No** | **Yes** | **No** | **Yes** | **No** | **Yes** |
| ***n*** | ***5,704*** | ***1,119*** | ***2,648*** | ***491*** | ***4,234*** | ***929*** | ***22,801*** | ***2,645*** | ***25,628*** | ***2,968*** |
| **Household size** | **ns** | | **ns** | | **ns** | | ******* | | ******* | |
| 1-3 | 80.8 | 19.3 | 85.3 | 14.7 | 81.9 | 18.1 | 89.1 | 10.9 | 89.1 | 11.0 |
| 4-6 | 84.2 | 15.8 | 85.6 | 14.4 | 82.5 | 17.5 | 90.9 | 9.1 | 91.0 | 9.0 |
| 7&+ | 83.6 | 16.4 | 83.0 | 17.1 | 81.6 | 18.4 | 88.5 | 11.5 | 88.5 | 11.5 |
| **Sex of the head of household** | **ns** | | ******* | | **ns** | | ******* | | ******* | |
| Male | 83.5 | 16.6 | 83.9 | 16.1 | 81.7 | 18.3 | 89.3 | 10.7 | 89.4 | 10.6 |
| Female | 85.5 | 14.5 | 89.2 | 10.8 | 85.2 | 14.8 | 92.7 | 7.3 | 91.6 | 8.4 |
| **Age of the mother** | ******* | | **ns** | | ***** | | ******* | | ******* | |
| 15-19 | 76.1 | 23.9 | 83.6 | 16.4 | 77.3 | 22.7 | 85.0 | 15.0 | 85.8 | 14.2 |
| 20-24 | 82.5 | 17.5 | 82.7 | 17.3 | 80.1 | 19.9 | 88.9 | 11.1 | 88.2 | 11.8 |
| 25-29 | 83.3 | 16.7 | 86.1 | 13.9 | 82.3 | 17.7 | 90.1 | 9.9 | 89.7 | 10.3 |
| 30-34 | 85.5 | 14.5 | 84.4 | 15.6 | 82.3 | 17.7 | 90.5 | 9.5 | 90.6 | 9.5 |
| 35-39 | 85.4 | 14.6 | 83.3 | 16.7 | 84.6 | 15.4 | 90.1 | 9.9 | 90.0 | 10.0 |
| 40-44 | 86.9 | 13.1 | 85.3 | 14.7 | 85.2 | 14.8 | 89.6 | 10.4 | 92.0 | 8.0 |
| 45-49 | 78.1 | 21.9 | 87.5 | 12.5 | 80.4 | 19.6 | 89.1 | 11.0 | 89.2 | 10.8 |
| **Place of residence** | ******* | | **ns** | | ******* | | ******* | | ******* | |
| Urban | 90.1 | 9.9 | 85.5 | 14.5 | 85.2 | 14.8 | 91.1 | 8.9 | 91.0 | 9.0 |
| Rural | 79.9 | 20.1 | 83.9 | 16.1 | 80.2 | 19.8 | 89.0 | 11.0 | 88.9 | 11.1 |
| **Mother Occupation** | ******* | | ******* | | **ns** | | ******* | | ******* | |
| Not working | 81.4 | 18.6 | 83.5 | 16.5 | 81.3 | 18.8 | 89.4 | 10.7 | 88.3 | 11.7 |
| Sale | 83.0 | 17.1 | 85.2 | 14.8 | 81.2 | 18.8 | 88.9 | 11.1 | 90.4 | 9.6 |
| Agriculter | 85.5 | 14.5 | 81.2 | 18.8 | 84.5 | 15.6 | 91.0 | 9.0 | 90.6 | 9.4 |
| Manual | 89.7 | 10.3 | 88.1 | 11.9 | 83.5 | 16.6 | 89.7 | 10.3 | 89.4 | 10.6 |
| **Mother's education level** | ******* | | ******* | | ******* | | ******* | | ******* | |
| No Education | 80.6 | 19.4 | 81.5 | 18.5 | 77.2 | 22.8 | 86.6 | 13.4 | 87.5 | 12.5 |
| 1-6 years | 85.4 | 14.6 | 86.0 | 14.0 | 84.7 | 15.4 | 90.9 | 9.2 | 89.9 | 10.1 |
| 7&+ years | 91.6 | 8.4 | 87.9 | 12.1 | 88.2 | 11.8 | 93.9 | 6.1 | 92.3 | 7.7 |
| **Has Electricity** | ******* | | ******* | | ******* | | ******* | | ******* | |
| No | 79.8 | 20.2 | 83.0 | 17.0 | 79.4 | 20.6 | 88.0 | 12.0 | 88.0 | 12.0 |
| Yes | 90.4 | 9.6 | 86.3 | 13.8 | 85.0 | 15.1 | 92.1 | 8.0 | 91.4 | 8.6 |
| **Has Television** | ******* | | ******* | | ******* | | ******* | | ******* | |
| No | 81.1 | 18.9 | 83.6 | 16.4 | 80.1 | 19.9 | 88.0 | 12.0 | 87.9 | 12.1 |
| Yes | 90.5 | 9.5 | 86.6 | 13.4 | 86.5 | 13.5 | 93.1 | 7.0 | 91.9 | 8.1 |
| **Has Refrigerator** | ******* | | ******* | | ******* | | ******* | | ******* | |
| No | 82.4 | 17.6 | 83.8 | 16.2 | 81.0 | 19.0 | 89.0 | 11.0 | 89.0 | 11.0 |
| Yes | 90.3 | 9.8 | 87.6 | 12.4 | 87.2 | 12.8 | 94.0 | 6.0 | 92.7 | 7.4 |
| **Has Bicycle** | ******* | | **ns** | | ******* | | ******* | | ******* | |
| No | 84.6 | 15.4 | 84.6 | 15.4 | 84.0 | 16.0 | 90.3 | 9.7 | 89.9 | 10.1 |
| Yes | 81.3 | 18.7 | 83.7 | 16.3 | 79.1 | 20.9 | 87.9 | 12.1 | 88.7 | 11.3 |
| **Has Motorcycle** | **0.324** | | **0.2** | | **0.043** | | **0.001** | | **0.066** | |
| No | 83.4 | 16.6 | 84.7 | 15.3 | 81.5 | 18.5 | 89.2 | 10.8 | 89.9 | 10.1 |
| Yes | 84.5 | 15.5 | 82.5 | 17.5 | 84.2 | 15.8 | 90.5 | 9.5 | 89.2 | 10.8 |
| ***Immediat environment and hygiene*** |  |  |  |  |  |  |  |  |  |  |
| **Type of toilette** | ******* | | ******* | | ******* | | ******* | | ******* | |
| Flush/upgraded latrin | 94.4 | 5.6 | 88.5 | 11.5 | 90.2 | 9.8 | 95.6 | 4.4 | 93.4 | 6.6 |
| Traditional latrines | 83.0 | 17.0 | 83.0 | 17.0 | 80.2 | 19.9 | 87.4 | 12.6 | 88.3 | 11.7 |
| Lack of toilet | 80.3 | 19.7 | 86.6 | 13.4 | 82.8 | 17.2 | 90.5 | 9.5 | 89.3 | 10.7 |
| Other type | 81.6 | 18.4 | 77.2 | 22.8 | 85.1 | 14.9 | 95.0 | 5.0 | 95.6 | 4.5 |
| **Quality of Main floor material** | ******* | | ******* | | ******* | | ******* | | ******* | |
| Poor | 81.3 | 18.7 | 84.5 | 15.5 | 77.4 | 22.6 | 86.7 | 13.3 | 87.6 | 12.4 |
| Middle | 85.3 | 14.7 | 84.0 | 16.0 | 82.9 | 17.1 | 91.3 | 8.7 | 90.9 | 9.1 |
| Rich | 86.1 | 13.9 | 96.7 | 3.3 | 89.7 | 10.3 | 95.0 | 5.0 | 92.0 | 8.0 |
| **Sources of drinking water** | ******* | | **ns** | | ******* | | ******* | | ******* | |
| Piped/Public tap/stan | 88.0 | 12.0 | 86.2 | 13.8 | 84.7 | 15.3 | 90.1 | 9.9 | 88.6 | 11.4 |
| Tube well or borehole | 79.4 | 20.6 | 83.1 | 16.9 | 77.7 | 22.3 | 90.7 | 9.3 | 91.2 | 8.8 |
| Well | 82.7 | 17.3 | 84.4 | 15.6 | 87.5 | 12.5 | 87.6 | 12.4 | 88.5 | 11.5 |
| Spring/river/lake/other | 91.6 | 8.4 | 84.3 | 15.7 | 81.8 | 18.2 | 91.0 | 9.0 | 89.4 | 10.6 |
| ***Child level variables*** |  |  |  |  |  |  |  |  |  |  |
| **Sex of the child** | ******* | | **ns** | | ***** | | ****** | | **ns** | |
| Female | 84.8 | 15.2 | 84.2 | 15.8 | 83.1 | 16.9 | 90.1 | 9.9 | 89.7 | 10.4 |
| Male | 82.4 | 17.6 | 84.5 | 15.5 | 81.0 | 19.0 | 89.1 | 10.9 | 89.6 | 10.4 |
| **Age in months** | ******* | | ******* | | ******* | | ****** | | ******* | |
| 0-6 | 87.0 | 13.0 | 90.6 | 9.4 | 84.6 | 15.4 | 92.0 | 8.0 | 92.8 | 7.2 |
| 07-12 | 72.4 | 27.6 | 80.1 | 19.9 | 74.0 | 26.0 | 83.4 | 16.6 | 82.6 | 17.4 |
| 13-18 | 71.8 | 28.2 | 81.0 | 19.1 | 71.3 | 28.7 | 82.8 | 17.2 | 83.3 | 16.7 |
| 19-24 | 77.1 | 23.0 | 82.0 | 18.0 | 71.8 | 28.3 | 86.4 | 13.6 | 85.1 | 14.9 |
| 25-30 | 80.8 | 19.2 | 86.2 | 13.8 | 78.9 | 21.1 | 88.5 | 11.5 | 89.0 | 11.0 |
| 31-36 | 84.3 | 15.7 | 86.8 | 13.3 | 83.0 | 17.0 | 91.8 | 8.2 | 90.0 | 10.0 |
| 37-42 | 90.9 | 9.1 | - | - | 86.1 | 13.9 | 91.4 | 8.6 | 92.6 | 7.4 |
| 42-48 | 91.3 | 8.7 | - | - | 90.2 | 9.8 | 93.8 | 6.2 | 93.5 | 6.5 |
| 48-59 | 92.1 | 8.0 | - | - | 92.9 | 7.1 | 94.4 | 5.6 | 94.8 | 5.2 |
| **Has Measles vaccine** | ******* | | **ns** | | ******* | | ******* | | ******* | |
| No | 81.3 | 18.7 | 83.9 | 16.1 | 79.0 | 21.0 | 88.0 | 12.1 | 88.1 | 11.9 |
| Yes | 86.6 | 13.4 | 85.3 | 14.8 | 87.9 | 12.2 | 92.5 | 7.5 | 92.1 | 8.0 |
| **Size of child at birth** | ******* | | **ns** | | ******* | | ******* | | ******* | |
| Large | 85.9 | 14.1 | 85.4 | 14.6 | 85.1 | 14.9 | 89.9 | 10.2 | 89.3 | 10.7 |
| Average | 83.4 | 16.6 | 83.9 | 16.1 | 80.9 | 19.1 | 90.5 | 9.5 | 91.1 | 8.9 |
| Small | 79.9 | 20.1 | 83.9 | 16.1 | 76.4 | 23.6 | 86.0 | 14.1 | 85.9 | 14.1 |
| DK (Don't know) | 75.6 | 24.4 | 79.1 | 20.9 | 69.7 | 30.3 | 94.3 | 5.7 | 95.6 | 4.4 |

**=p-value<0.05; **=p-value <0.01; ***=p-value <0.001, ns= no significant*

Appendix 1: Determinant Variables in Bivariate Analysis (Count…)

|  | **Niger** | | | | | | | |
| --- | --- | --- | --- | --- | --- | --- | --- | --- |
| ***Variable*** | 1992 | | 1998 | | 2006 | | 2012 | |
|  | **No** | **Yes** | **No** | **Yes** | **No** | **Yes** | **No** | **Yes** |
| ***n*** | ***4,168*** | ***1,424*** | ***2,700*** | ***1,543*** | ***6,540*** | ***1,669*** | ***10,011*** | ***1,591*** |
| **Household size** | ****** | | **ns** | | ****** | | ******* | |
| 1-3 | 67.0 | 33.0 | 64.0 | 36.0 | 74.7 | 25.3 | 80.1 | 19.9 |
| 4-6 | 74.4 | 25.6 | 64.8 | 35.3 | 80.0 | 20.0 | 86.1 | 13.9 |
| 7&+ | 75.3 | 24.7 | 63.0 | 37.1 | 80.1 | 19.9 | 87.2 | 12.8 |
| **Sex of the head of household** | **ns** | | **ns** | | **ns** | | **ns** | |
| Male | 74.6 | 25.4 | 63.4 | 36.7 | 80.0 | 20.0 | 86.3 | 13.7 |
| Female | 73.0 | 27.0 | 67.1 | 32.9 | 77.7 | 22.3 | 86.2 | 13.8 |
| **Age of the mother** | ****** | | **ns** | | ******* | | ******* | |
| 15-19 | 66.8 | 33.2 | 64.6 | 35.4 | 73.9 | 26.1 | 80.2 | 19.8 |
| 20-24 | 75.5 | 24.5 | 63.6 | 36.4 | 77.7 | 22.3 | 85.7 | 14.3 |
| 25-29 | 75.1 | 24.9 | 65.0 | 35.0 | 79.5 | 20.5 | 86.5 | 13.5 |
| 30-34 | 76.0 | 24.0 | 64.0 | 36.1 | 81.8 | 18.2 | 86.9 | 13.1 |
| 35-39 | 72.9 | 27.1 | 62.5 | 37.5 | 82.6 | 17.4 | 86.7 | 13.3 |
| 40-44 | 75.2 | 24.8 | 59.6 | 40.4 | 79.5 | 20.5 | 88.5 | 11.5 |
| 45-49 | 79.4 | 20.6 | 53.2 | 46.8 | 79.9 | 20.1 | 89.8 | 10.2 |
| **Place of residence** | ******* | | ******* | | ******* | | ******* | |
| Urban | 80.4 | 19.6 | 68.7 | 31.3 | 82.9 | 17.1 | 84.6 | 15.4 |
| Rural | 70.6 | 29.4 | 61.9 | 38.1 | 78.3 | 21.7 | 86.8 | 13.2 |
| **Mother Occupation** | ******* | | ******* | | ******* | | **ns** | |
| Not working | 75.8 | 24.2 | 66.2 | 33.8 | 80.9 | 19.1 | 86.6 | 13.4 |
| Sale | 76.8 | 23.2 | 59.7 | 40.3 | 79.9 | 20.2 | 86.3 | 13.7 |
| Agriculter | 67.5 | 32.5 | 60.6 | 39.4 | 74.9 | 25.2 | 82.5 | 17.5 |
| Manual | 71.2 | 28.8 | 68.2 | 31.8 | 80.2 | 19.8 | 84.5 | 15.5 |
| **Mother's education level** | ******* | | ******* | | ******* | | ******* | |
| **No Education** | 73.5 | 26.5 | 62.7 | 37.3 | 78.7 | 21.3 | 86.8 | 13.2 |
| 1-6 years | 77.8 | 22.2 | 65.9 | 34.1 | 82.6 | 17.4 | 84.1 | 15.9 |
| 7&+ years | 85.9 | 14.1 | 75.3 | 24.7 | 87.8 | 12.2 | 83.1 | 16.9 |
| **Has Electricity** | ******* | | ******* | | ******* | | ***** | |
| No | 73.8 | 26.2 | 62.6 | 37.4 | 79.0 | 21.1 | 86.7 | 13.3 |
| Yes | 80.5 | 19.5 | 72.8 | 27.2 | 83.4 | 16.6 | 84.8 | 15.3 |
| **Has Television** | ******* | | ******* | | ******* | | ******* | |
| No | 73.6 | 26.5 | 63.0 | 37.0 | 79.0 | 21.1 | 86.6 | 13.4 |
| Yes | 82.4 | 17.6 | 72.3 | 27.7 | 84.8 | 15.2 | 84.6 | 15.4 |
| **Has Refrigerator** | ****** | | ******* | | ******* | | **ns** | |
| No | 74.2 | 25.9 | 63.2 | 36.8 | 79.2 | 20.8 | 86.3 | 13.7 |
| Yes | 81.0 | 19.1 | 75.0 | 25.0 | 88.2 | 11.8 | 86.2 | 13.9 |
| **Has Bicycle** | **ns** | | **ns** | | ******* | | **ns** | |
| No | 74.3 | 25.7 | 63.9 | 36.1 | 79.0 | 21.1 | 86.3 | 13.7 |
| Yes | 76.6 | 23.4 | 60.6 | 39.4 | 83.6 | 16.4 | 86.5 | 13.5 |
| **Has Motorcycle** | ****** | | ******* | | **ns** | | **ns** | |
| No | 74.1 | 25.9 | 63.1 | 36.9 | 79.4 | 20.6 | 86.4 | 13.6 |
| Yes | 80.2 | 19.8 | 73.1 | 26.9 | 82.0 | 18.1 | 85.5 | 14.5 |
| ***Immediat environment and hygiene*** |  |  |  |  |  |  |  |  |
| **Type of toilette** | ****** | | **ns** | | ******* | | ***** | |
| Flush/upgraded latrin | 83.3 | 16.7 | 71.2 | 28.9 | 87.7 | 12.3 | 86.5 | 13.5 |
| Traditional latrines | 80.7 | 19.3 | 69.0 | 31.0 | 82.5 | 17.5 | 84.9 | 15.1 |
| Lack of toilet | 71.4 | 28.6 | 62.0 | 38.0 | 78.4 | 21.6 | 86.9 | 13.1 |
| Other type | 72.1 | 27.9 | 35.3 | 64.7 | 78.5 | 21.5 | 82.0 | 18.0 |
| **Quality of Main floor material** | ******* | | ******* | | ******* | | ******* | |
| Poor | 71.9 | 28.1 | 61.7 | 38.3 | 78.5 | 21.5 | 87.0 | 13.1 |
| Middle | 80.3 | 19.7 | 71.2 | 28.8 | 83.7 | 16.3 | 86.1 | 13.9 |
| Rich | 84.4 | 15.6 | 75.8 | 24.2 | 88.9 | 11.1 | 82.0 | 18.0 |
| **Sources of drinking water** | ****** | | ******* | | ******* | | ******* | |
| Piped/Public tap/stan | 78.2 | 21.8 | 68.8 | 31.2 | 82.5 | 17.6 | 85.1 | 14.9 |
| Tube well or borehole | 70.8 | 29.2 | 61.9 | 38.1 | - | - | 85.8 | 14.2 |
| Well | 76.9 | 23.1 | 65.6 | 34.4 | 78.2 | 21.8 | 87.6 | 12.4 |
| Spring/river/lake/other | 83.2 | 16.8 | 63.6 | 36.4 | 82.4 | 17.6 | 79.9 | 20.1 |
| ***Child level variables*** |  |  |  |  |  |  |  |  |
| **Sex of the child** | **ns** | | **ns** | | **ns** | | **ns** | |
| Female | 75.2 | 24.8 | 64.4 | 35.6 | 79.4 | 20.6 | 86.7 | 13.3 |
| Male | 73.9 | 26.1 | 62.9 | 37.1 | 79.9 | 20.1 | 85.8 | 14.2 |
| **Age in months** | ******* | | ******* | | ******* | | ******* | |
| 0-6 | 74.1 | 26.0 | 69.3 | 30.7 | 80.7 | 19.3 | 84.6 | 15.4 |
| 07-12 | 55.4 | 44.6 | 50.4 | 49.6 | 66.7 | 33.3 | 71.2 | 28.8 |
| 13-18 | 62.0 | 38.0 | 61.8 | 38.2 | 67.8 | 32.2 | 77.5 | 22.5 |
| 19-24 | 63.8 | 36.2 | 63.5 | 36.5 | 72.9 | 27.1 | 82.3 | 17.7 |
| 25-30 | 73.1 | 26.9 | 66.6 | 33.4 | 78.1 | 21.9 | 84.9 | 15.2 |
| 31-36 | 79.3 | 20.7 | 72.9 | 27.1 | 81.7 | 18.3 | 89.7 | 10.3 |
| 37-42 | 85.1 | 14.9 |  |  | 87.2 | 12.8 | 92.3 | 7.7 |
| 42-48 | 86.4 | 13.7 |  |  | 87.8 | 12.2 | 93.7 | 6.3 |
| 48-59 | 88.5 | 11.5 |  |  | 90.2 | 9.8 | 96.2 | 3.8 |
| **Has Measles vaccine** | ******* | | **ns** | | ******* | | ******* | |
| No | 71.7 | 28.3 | 62.7 | 37.3 | 77.3 | 22.7 | 84.1 | 15.9 |
| Yes | 79.3 | 20.7 | 65.8 | 34.2 | 82.4 | 17.6 | 87.7 | 12.3 |
| **Size of child at birth** | ******* | | **ns** | | ******* | | ******* | |
| Large | 78.2 | 21.8 | 63.1 | 36.9 | 79.6 | 20.4 | 85.5 | 14.5 |
| Average | 76.2 | 23.8 | 64.5 | 35.5 | 81.4 | 18.6 | 87.5 | 12.5 |
| Small | 69.5 | 30.5 | 62.5 | 37.5 | 75.5 | 24.5 | 83.5 | 16.5 |
| DK (Don't know) | 71.9 | 28.1 | 80.0 | 20.0 | 82.8 | 17.2 | 90.0 | 10.0 |

**=p-value<0.05; **=p-value <0.01; ***=p-value <0.001, ns= no significant*

Table 6: Multilevel Logistic Regression

|  | **Burkina Faso** | | | |
| --- | --- | --- | --- | --- |
|  | **1992-93** | **1998-99** | **2003** | **2010** |
| ***Variable*** | **AOR (95% CI)** | **AOR (95% CI)** | **AOR (95% CI)** | **AOR (95% CI)** |
| ***n*** |  |  |  |  |
| **Household size** |  |  |  |  |
| 1-3 | 1 | 1 | 1 | 1 |
| 4-6 | 1.1 (0.8; 1.6) | 0.9 (0.7; 1.3) | 0.7** (0.6; 0.9) | 0.8 (0.7;1.0) |
| 7&+ | 1.1 (0.8; 1.5) | 1.1 (0.8; 1.6) | 0.8*(0.6; 0.9) | 0.9 (0.7; 1.1) |
| **Sex of the head of household** |  |  |  |  |
| Male | 1 | 1 | 1 | 1 |
| Female | 0.8 | 0.9 (0.6; 1.3) | 1.2 (0.9; 1.5) | 0.9 (0.8; 1.2) |
| **Age of the mother** |  |  |  |  |
| 15-19 | 1 | 1 | 1 | 1 |
| 20-24 | 1.0 (0.7; 1.4) | 1.1 (0.8; 1.5) | 1.1 (0.8; 1.4) | 0.9 (0.7; 1.2) |
| 25-29 | 1.0 (0.7; 1.4) | 1.0 (0.7; 1.4) | 1.1 (0.8; 1.3) | 0.9(0.7; 1.1) |
| 30-34 | 1.0 (0.7; 1.4) | 0.9 (0.6; 1.3) | 1.1 (0.8; 1.4) | 0.9 (0.7; 1.2) |
| 35-39 | 1.0 (0.7; 1.4) | 1.0 (0.7; 1.4) | 1.1 (0.8; 1.4) | 0.9 (0.7; 1.2) |
| 40-44 | 1.1 (0.8; 1.7) | 0.8 (0.5; 1.3) | 1.0 (0.8; 1.4) | 1.0(0.7; 1.3) |
| 45-49 | 1.5 (0.9; 2.7) | 1.4 (0.8; 2.4) | 1.4 (0.9; 2.1) | 0.8 (0.5; 1.3) |
| **Place of residence** |  |  |  |  |
| Urban | 1 | 1 | 1 | 1 |
| Rural | 0.9 (0.7; 1.3) | 1.1 (0.7; 1.7) | 1.1 (0.8;1.4) | 1.0 (0.8; 1.3) |
| **Mother Occupation** |  |  |  |  |
| Not working | 1 | 1 | 1 | 1 |
| Sale | 1.2 (0.9; 1.4) | 1.3*(1.03; 1.6) | 0.9 (0.7; 1.1) | 1.2*(1.02; 1.5) |
| Agriculter | 1.2 (0.9; 1.6) | 1.4** (1.1; 1.7) | 0.8 (0.6; 1.0) | 1.2*(1.02; 1.4) |
| Manual | 1.1 (0.8; 1.4) | 1.3 (0.9; 1.7) | 1.0 (0.7; 1.5) | 1.4**(1.1; 1.7) |
| **Mother's education level** |  |  |  |  |
| No Education | 1 | 1 | 1 | 1 |
| 1-6 years | 1.2 (0.9; 1.6) | 1.1 (0.8; 1.4) | 1.1 (0.9; 1.3) | 1.2(1.0; 1.4) |
| 7&+ years | 0.9 (0.6; 1.3) | 0.9 (0.5; 1.4) | 0.8 (0.5; 1.1) | 1.0 (0.8; 1.3) |
| **Has Electricity** |  |  |  |  |
| No | 0.9 (0.6; 1.4) | 0.5* (0.3; 0.8) | 0.9 (0.7; 1.3) | 0.9 (0.7; 1.3) |
| Yes | 1 | 1 | 1 | 1 |
| **Has Television** |  |  |  |  |
| No | 0.9 (0.6; 1.4) | 0.9 (0.6; 1.4) | 0.9 (0.6; 1.13) | 0.9 (0.8; 1.1) |
| Yes | 1 | 1 | 1 | 1 |
| **Has Refrigerator** |  |  |  |  |
| No | 0.9 (0.5; 1.6) | 1.6 (0.9; 3.1) | 0.7 (0.5; 1.1) | 0.8 (0.5; 1.2) |
| Yes | 1 | 1 | 1 | 1 |
| **Has Bicycle** |  |  |  |  |
| No | 0.9 (0.8; 1.2) | 0.8* (0.6; 0.9) | 0.9 (0.8; 1.1) | 0.8 (0.7; 1.0) |
| Yes | 1 | 1 | 1 | 1 |
| **Has Motorcycle** |  |  |  |  |
| No | 0.9 (0.8; 1.13) | 0.9 (0.8; 1.2) | 0.9 (0.8; 1.1) | 0.9 (0.8; 1.1) |
| Yes | 1 | 1 | 1 | 1 |
| ***Immediat environment and hygiene*** |  |  |  |  |
| **Type of toilette** |  |  |  |  |
| Flush/upgraded latrin | 1 | 1 | 1 | 1 |
| Traditional latrines | 0.9 (0.4; 2.5) | 0.8 (0.3; 2.3) | 0.6 (0.3; 1.0) | 0.6 (0.4; 1.0) |
| Lack of toilet | 0.9 (0.4; 2.4) | 0.6 (0.2; 1.9) | 0.5(0.3; 0.9) | 0.6*(0.3; 0.9) |
| Other type | 0.9 (0.3; 3.4) | 0.9 (0.3; 3.3) | 0.4 (0.2; 9) | 0.4*(0.2; 0.9) |
| **Quality of Main floor material** |  |  |  |  |
| Poor | 1 | 1 | 1 | 1 |
| Middle | 0.7** (0.6; 0.9) | 1.0 (0.8; 1.2) | 0.9 (0.8; 1.1) | 1.1 (0.9; 1.2) |
| Rich | 0.9 (0.4; 2.6) | 1.9 (0.8; 4.8) | 1.3 (0.7; 2.3) | 0.9 (0.5; 1.8) |
| **Sources of drinking water** |  |  |  |  |
| Piped/Public tap/stan | 1 | 1 | 1 | 1 |
| Tube well or borehole | 0.8 (0.6; 1.1) | 0.9 (0.6; 1.3) | 0.9 (0.8; 1.1) | 0.9 (0.8; 1.2) |
| Well | 0.7 (0.4; 1.2) | 0.8 (0.5; 1.4) | 0.9 (0.7; 1.2) | 1.0 (0.8; 1.2) |
| Spring/river/lake/other | 1.1 (0.7; 1.8) | 0.7 (0.3; 1.4) | 0.9 (0.7; 1.2) | 1.0 (0.8; 1.4) |
| ***Child level variables*** |  |  |  |  |
| **Sex of the child** |  |  |  |  |
| Female | 1 | 1 | 1 | 1 |
| Male | 1.2**(1.1; 1.4) | 1.1 (0.9; 1.2) | 1.2** (1.1; 1.3) | 1.0 (0.9; 1.1) |
| **Age in months** |  |  |  |  |
| 0-6 | 1 | 1 | 1 | 1 |
| 07-12 | 2.0***(1.5; 2.6) | 2.8***(2.2; 3.8) | 2.7***(2.2; 3.3) | 2.7***(2.2; 3.3) |
| 13-18 | 2.8***(2.1; 3.7) | 2.8** (2.1; 3.8) | 2.9***(2.3; 3.6) | 3.2***(2.6; 4.1) |
| 19-24 | 1.7***(1.2; 2.4) | 2.5*** (1.8; 3.4) | 2.5***(1.9; 3.1) | 2.3***(1.8; 3.0) |
| 25-30 | 1.6***(1.2; 2.2) | 2.1*** (1.6; 2.9) | 2.2***(1.7; 2.8) | 2.0***(1.6; 2.6) |
| 31-36 | 1.3 (0.9; 1.8) | 1.7** (1.2; 2.3) | 1.3* (1.0 ; 1.7) | 1.4** (1.1; 1.9) |
| 37-42 | 0.81 (0.6; 1.1) | 1.1 (0.8; 1.6) | 1.1 (0.8; 1.4) | 0.9 (0.7; 1.2) |
| 42-48 | 0.7 (0.5; 1.1) | 0.8 (0.6; 1.2) | 0.8 (0.9; 1.4) | 0.7*(0.5; 0.9) |
| 48-59 | 0.3***(0.2; 0.5) | 0.5*** (0.3; 0.7) | 0.5*** (0.4; 0.7) | 0.5***(0.4; 0.6) |
| **Has Measles vaccine** |  |  |  |  |
| No | 0.9 (0.8; 1.1) | 0.9 (0.8; 1.1) | 0.9*(0.8; 0.9) | 0.9(0.8; 1.1) |
| Yes | 1 | 1 | 1 | 1 |
| **Size of child at birth** |  |  |  |  |
| Large | 1 | 1 |  | 1 |
| Average | 0.9 (0.8; 1.1) | 0.9 (0.7; 1.0) | 0.8**(0.7; 0.9) | 0.8***(0.7; 0.9) |
| Small | 1.3**(1.1; 1.5) | 1.0 (0.8; 1.2) | 1.0 (0.8; 1.2) | 1.0(0.9; 1.2) |
| DK (Don't know) | 0.9 (0.6; 1.6) | 0.3 (0.04; 2.4) | 0.5*(0.3; 0.9) | 0.5(0.2; 1.4) |
|  |  |  |  |  |
| ***Constant*** | 0.2* (0.1; 0.6) | 0.2* (0.1; 0.8) | 0.6 (0.3; 1.1) | 0.3**(.1; 0.5) |
| ***Community Level SD*** | 0.39 (0.29; 0.54) | 0.46 (0.36 ; 0.58) | 0.50 (0.43; 0.59) | 0.56 (0.49; 0.65) |
| ***ICC*** | 0.04 (0.2; 0.08) | 0.06 (0.04; 0.09) | 0.07 (0.05; 0.10) | 0.09 (0.07; 0.11) |

Appendix 2: Multilevel Logistic Regression (Count…)

|  | **Mali** | | | |
| --- | --- | --- | --- | --- |
|  | **1995** | **2001** | **2006** | **2012** |
| ***Variable*** | **AOR (95% CI)** | **AOR (95% CI)** | **AOR (95% CI)** | **AOR (95% CI)** |
| ***n*** |  |  |  |  |
| **Household size** |  |  |  |  |
| 1-3 | 1 | 1 | 1 | 1 |
| 4-6 | 1.2 (0.9; 1.6) | 0.8*(0.7; 0.9) | 0.9 (0.7; 1.2) | 1.0 (0.7; 1.4) |
| 7&+ | 1.1 (0.9; 1.5) | 0.8*(0.7; 0.9) | 0.9 (0.7; 1.2) | 0.9 (0.7; 1.3) |
| **Sex of the head of household** |  |  |  |  |
| Male | 1 | 1 | 1 | 1 |
| Female | 1.04 (0.8; 1.4) | 0.9(0.7; 1.1) | 1.2(0.9; 1.5) | 1.0 (0.7; 1.4) |
| **Age of the mother** |  |  |  |  |
| 15-19 | 1 | 1 | 1 | 1 |
| 20-24 | 1.04 (0.8; 1.3) | 0.9(0.8; 1.1) | 1.0 (0.8; 1.3) | 0.9 90.7; 1.2) |
| 25-29 | 1.0 (0.8; 1.3) | 0.9(0.7; 1.1) | 1.1 (0.8; 1.4) | 0.8 (0.6; 1.1) |
| 30-34 | 1.1 (0.9; 1.5) | 1.0(0.8; 1.3) | 0.9 (0.8; 1.3) | 0.8 (0.6; 1.1) |
| 35-39 | 1.1 (0.8; 1.5) | 0.9 (0.8; 1.2) | 0.9 (0.7; 1.3) | 0.9 (0.7; 1.4) |
| 40-44 | 1.3(0.9; 1.8) | 1.1(0.9; 1.5) | 0.9 (0.7; 1.3) | 0.9 (0.6; 1.3) |
| 45-49 | 1.6 (0.9; 2.8) | 0.8(0.5; 1.2) | 1.1(0.7; 1.7) | 0.6 (0.3; 1.2) |
| **Place of residence** |  |  |  |  |
| Urban | 1 | 1 | 1 | 1 |
| Rural | 0.9 (0.8; 1.2) | 1.3*(1.1; 1.6) | 1.5**(1.1; 1.9) | 1.1 (0.8; 1.6) |
| **Mother Occupation** |  |  |  |  |
| Not working | 1 |  | 1 | 1 |
| Sale | 1.3**(1.1; 1.5) | 1.1(0.9; 1.2) | 1.3*(1.1; 1.5) | 1.8***(1.5; 2.3) |
| Agriculter | 1.2*(1.03; 1.5) | 1.1(0.9; 1.3) | - | 1.8***(1.4; 2.3) |
| Manual | 1.1 (0.8; 1.4) | 1.3**(1.1; 1.6) | 1.4***(1.2;1.6) | 1.8***(1.4; 2.3) |
| **Mother's education level** |  |  |  |  |
| No Education | 1 | 1 | 1 | 1 |
| 1-6 years | 0.7 (0.6; 0.9) | 0.9 (0.8; 1.2) | 1.02 (0.8;1.3) | 1.2 (0.9; 1.6) |
| 7&+ years | 0.6*(0.4; 0.9) | 1.1(10.8; 1.5) | 1.1 (0.8; 1.5) | 1.3 (0.9; 1.7) |
| **Has Electricity** |  |  |  |  |
| No | 0.9 (0.6; 1.4) | 1.0 (0.8; 1.3) | 0.8 (0.6; 1.1) | 1.1 (0.8; 1.4) |
| Yes | 1 | 1 | 1 | 1 |
| **Has Television** |  |  |  |  |
| No | 0.7 (0.5; 1.02) | 0.9(0.7; 1.1) | 0.9 90.7; 1.04) | 0.9 (0.7; 1.1) |
| Yes | 1 | 1 | 1 | 1 |
| **Has Refrigerator** |  |  |  |  |
| No | 0.9(0.5; 1.5) | 1.1 (0.8; 1.6) | 1.3 (0.8; 1.9) | 1.0 (0.7; 1.5) |
| Yes | 1 | 1 | 1 | 1 |
| **Has Bicycle** |  |  |  |  |
| No | 0.9(0.8; 1.05) | 0.8**(0.7; 0.9) | 1.0 (0.9; 1.2) | 0.9 (0.8; 1.2) |
| Yes | 1 | 1 | 1 | 1 |
| **Has Motorcycle** |  |  |  |  |
| No | 1.2(0.9; 1.4) | 1.0(0.9; 1.1) | 0.9 (0.8; 1.1) | 0.9 (0.8; 1.1) |
| Yes | 1 | 1 | 1 | 1 |
| ***Immediat environment and hygiene*** |  |  |  |  |
| **Type of toilette** |  |  |  |  |
| Flush/upgraded latrin | 1 | 1 | 1 | 1 |
| Traditional latrines | 1.7 (0.5; 5.1) | 1.4*(1.1; 1.9) | 1.4 (0.8; 2.4) | 1.2 (0.9; 1.7) |
| Lack of toilet | 1.9 (0.6; 6.1) | 1.7***(1.2; 2.3) | 1.4 (0.8; 2.4) | 1.5*(1.02; 2.3) |
| Other type | 0.6 (0.1; 3.7) | 1.2 (0.9; 1.1) | 1.1 (0.5; 2.5) | 1.3 (0.5; 3.1) |
| **Quality of Main floor material** |  |  |  |  |
| Poor | 1 | 1 | 1 | 1 |
| Middle | 0.6***(0.5; 0.8) | 0.8*(0.6; 0.9) | 0.9 (0.8; 1.2) | 1.1 (0.8; 1.3) |
| Rich | 0.9 (0.4; 2.2) | 1.1 (0.6; 1.7) | 1.1 (0.6; 1.9) | 1.3(0.8; 2.0) |
| **Sources of drinking water** |  |  |  |  |
| Piped/Public tap/stan | 1 | 1 | 1 | 1 |
| Tube well or borehole | 0.8 (0.7; 1.1) | 1.3**(1.1; 1.5) | - | 0.8 (0.6; 1.04) |
| Well | 1.3 (0.8; 2.0) | 1.2(0.9; 1.4) | 1.0 (0.8; 1.2) | 0.8 (0.6; 1.1) |
| Spring/river/lake/other | 1.3(0.3; 5.8) | 1.7***(1.3; 2.2) | 0.9 (0.7; 1.3) | 0.8(0.4; 1.3) |
| ***Child level variables*** |  |  |  |  |
| **Sex of the child** |  |  |  |  |
| Female | 1 | 1 | 1 | 1 |
| Male | 1.1 (0.9; 1.2) | 1.1 (0.9; 1.2) | 1.1 (0.9; 1.2) | 1.2(0.9; 1.3) |
| **Age in months** |  |  |  |  |
| 0-6 | 1 | 1 | 1 | 1 |
| 07-12 | 2.6*** (2.1; 3.2) | 2.4***(2.0; 2.9) | 3.7***(2.9; 4.7) | 2.0***(1.4; 2.7) |
| 13-18 | 2.9***(2.3; 3.7) | 2.2***(1.8; 2.6) | 3.6***(2.8; 4.7) | 1.8***(1.4; 2.5) |
| 19-24 | 2.4***(1.9; 3.1) | 2.0***(1.6; 2.5) | 3.6***(2.7; 4.7) | 1.3(0.9; 1.8) |
| 25-30 | 1.9***(1.5; 2.5) | 1.6***(1.3; 19) | 2.3***(1.7; 3.0) | 1.1 (0.8; 1.6) |
| 31-36 | 1.7***(1.3; 2.2) | 1.3*(0.1; 1.6) | 1.7***(1.3; 2.3) | 0.8 (0.6; 1.2) |
| 37-42 |  | 0.9 (0.7; 1.1) | 1.6**(1.2; 2.1) | 0.8 (0.5; 1.1) |
| 42-48 |  | 0.8(0.7; 1.0) | 0.8 (0.5; 1.1) | 0.6*(0.4; 0.8) |
| 48-59 |  | 0.5***(0.4; 0.7) | 0.7* (0.5; 0.9) | 0.6**(0.4; 0.8) |
| **Has Measles vaccine** |  |  |  |  |
| No | 1.0 (0.8; 1.2) | 0.9*(0.8; 0.9) | 0.9 (0.8; 1.0) | 1.2* (1.0; 1.5) |
| Yes | 1 | 1 | 1 | 1 |
| **Size of child at birth** |  |  |  |  |
| Large | 1 | 1 | 1 | 1 |
| Average | 0.9 (0.7; 1.0) | 0.9 (0.8; 1.0) | 1.0 (0.9; 1.1) | 0.9 (0.8; 1.1) |
| Small | 1.1 (0.8; 1.3) | 1.0 (0.9; 1.2) | 1.3***(1.1; 1.5) | 0.8(0.6; 1.1) |
| DK (Don't know) | 0.4*(0.2; 0.8) | 0.7 (0.5; 1) | 0.6 (0.4; 1.1) | 0.8(0.5; 1.2) |
|  |  |  |  |  |
| ***Constant*** | 0.10***(0.03; 0.33) | 0.12***(0.08; 0.2) | 0.03***(0.01; 0.05) | 0.04***(0.02; 0.1) |
| ***Community Level SD*** | 0.37 (0.28; 0.50) | 0.50(0.43; 0.59) | 0.70 (0.60; 0.80) | 0.80 (0.70; 0.93) |
| ***ICC*** | 0.04 (0.02; 0.07) | 0.07 (0.05; 0.10) | 0.13 (0.10; 0.16) | 0.16 (0.12; 0.21) |

Appendix 2: Multilevel Logistic Regression (Count…)

|  | **Nigeria** | | | | |
| --- | --- | --- | --- | --- | --- |
|  | **1990** | **1995** | **2001** | **2006** | **2012** |
| ***Variable*** | **AOR (95% CI)** | **AOR (95% CI)** | **AOR (95% CI)** | **AOR (95% CI)** | **AOR (95% CI)** |
| ***n*** |  |  |  |  |  |
| **Household size** |  |  |  |  |  |
| 1-3 | 1 | 1 | 1 | 1 | 1 |
| 4-6 | 0.9 (0.7;1.2) | 1.0 (0.7; 1.5) | 1.1 (0.8; 1.4) | 0.8 (0.7;1.0) | 0.9 (0.8; 1.1) |
| 7&+ | 0.9 (0.7; 1.2) | 1.2 (0.8; 1.7) | 1.0 (0.7; 1.3) | 0.9 (0.8; 1.1) | 1.1 (0.9; 1.3) |
| **Sex of the head of household** |  |  |  |  |  |
| Male | 1 | 1 | 1 | 1 | 1 |
| Female | 1.1 (0.8; 1.5) | 0.7 (0.4; 1.1) | 1.1 (0.8; 1.6) | 0.9 (0.7; 1.1) | 0.9 (0.8; 1.1) |
| **Age of the mother** |  |  |  |  |  |
| 15-19 | 1 | 1 | 1 | 1 | 1 |
| 20-24 | 0.8 (0.6: 1.2) | 1.1 (0.7; 1.6) | 1.0 (0.7; 1.4) | 0.9 (0.8; 1.1) | 0.9 (0.8; 1.2) |
| 25-29 | 0.9 (0.7;1.2) | 0.8 (0.6; 1.3) | 0.9 (0.7; 1.4) | 0.9 (0.8; 1.1) | 0.9 (0.8; 1.1) |
| 30-34 | 0.8 (0.6: 1.1) | 0.9 (0.6; 1.5) | 0.9 (0.7; 1.4) | 0.9 (0.7; 1.1) | 0.9 (0.7; 1.1) |
| 35-39 | 0.8 (0.5; 1.1) | 0.9 (0.6; 1.5) | 0.8 (0.6; 1.3) | 0.9 (0.7; 1.1) | 0.9 (0.7; 1.1) |
| 40-44 | 0.6* (0.4; 0.9) | 0.7 (0.4: 1.4) | 0.9 (0.5; 1.4) | 0.9 (0.7; 1.2) | 0.7*(0.5; 0.9) |
| 45-49 | 1.1 (0.7; 1.8) | 0.5 (0.2; 1.5) | 1.4 (0.7; 2.7) | 0.8 (0.6; 1.1) | 0.9 (0.7; 1.3) |
| **Place of residence** |  |  |  |  |  |
| Urban | 1 | 1 | 1 | 1 | 1 |
| Rural | 1.6**(1.2; 2.3) | 0.9 (0.7; 1.3) | 1.2 (0.9; 1.6) | 1.1 (0.9; 1.3) | 0.9 (0.8; 1.1) |
| **Mother Occupation** |  |  |  |  |  |
| Not working | 1 | 1 | 1 | 1 | 1 |
| Sale | 1.3**(1.1; 1.6) | 1.1 (0.9; 1.5) | 1.2* (1.0; 1.5) | 1.2 (1.1; 1.4) | 1.1 (0.9; 1.2) |
| Agriculter | 0.8* (0.6; 0.9) | 1.6* (1.1; 2.4) | 0.9 (0.7; 1.3) | 1.03 (0.9; 1.2) | 1.1 (0.9; 1.3) |
| Manual | 1.0 (0.7; 1.4) | 0.9 (0.6; 1.3) | 1.2 (0.9; 1.6) | 1.1 (0.9; 1.3) | 1.2* (1.1; 1.3) |
| **Mother's education level** |  |  |  |  |  |
| No Education | 1 | 1 | 1 | 1 | 1 |
| 1-6 years | 1.0 (0.8; 1.2) | 0.7 (0.5; 1.0) | 0.8* (0.6; 0.9) | 0.9 (0.9; 1.1) | 1.0 (0.9; 1.2) |
| 7&+ years | 0.7* (0.5; 0.9) | 0.7* (0.5; 0.9) | 0.7**(0.5; 0.9) | 0.8* (0.7; 0.9) | 0.9 (0.8; 1.01) |
| **Has Electricity** |  |  |  |  |  |
| No | 0.7* (0.5; 0.9) | 0.8(0.6; 1.1) | 1.0 (0.8; 1.3) | 0.8* (0.7; 0.9) | 0.9 (0.8; 1.1) |
| Yes | 1 | 1 | 1 | 1 | 1 |
| **Has Television** |  |  |  |  |  |
| No | 0.8 (0.6; 1.2) | 1.1 (0.7; 1.5) | 1.0 (0.8; 1.3) | 0.9* (0.7; 1.0) | 0.9 (0.8; 1.0) |
| Yes | 1 | 1 | 1 | 1 | 1 |
| **Has Refrigerator** |  |  |  |  |  |
| No | 1.4* (1.0; 1.9) | 0.9 (0.6; 1.4) | 1.0 (0.8; 1.4) | 0.9 (0.8; 1.2) | 1.0 (0.8; 1.2) |
| Yes | 1 | 1 | 1 | 1 | 1 |
| **Has Bicycle** |  |  |  |  |  |
| No | 1.1 (0.9; 1.3) | 0.9 (0.7; 1.2) | 1.2 (0.9; 1.4) | 1.0 (0.9; 1.1) | 0.9 (0.9; 1.1) |
| Yes | 1 | 1 | 1 | 1 | 1 |
| **Has Motorcycle** |  |  |  |  |  |
| No | 0.9 (0.7; 1.1) | 1.2 (0.9; 1.7) | 0.9 (0.8; 1.2) | 0.9 (0.9; 1.1) | 1.1 (1.01; 1.2) |
| Yes | 1 | 1 | 1 | 1 | 1 |
| ***Immediat environment and hygiene*** |  |  |  |  |  |
| **Type of toilette** |  |  |  |  |  |
| Flush/upgraded latrin | 1 | 1 | 1 | 1 | 1 |
| Traditional latrines | 1.8**(1.2; 2.8) | 1.1 (0.7; 1.8) | 1.3 (0.9; 1.9) | 1.6*** (1.2; 2.0) | 1.3**(1.1; 1.5) |
| Lack of toilet | 2.1**(1.3; 3.3) | 0.8 (0.5; 1.4) | 1.0 (0.7; 1.6) | 1.2 (0.9; 1.6) | 1.2 (0.9; 1.5) |
| Other type | 2.9* (1.2; 7.3) | 1.7 (0.7; 3.9) | 1.2 (0.5; 2.6) | 0.7 (0.5; 1.1) | 0.7* (0.4; 1.0) |
| **Quality of Main floor material** |  |  |  |  |  |
| Poor | 1 | 1 | 1 | 1 | 1 |
| Middle | 1.1 (0.9; 1.3) | 1.2 (0.9; 1.5) | 0.9 (0.7; 1.1) | 0.8*(0.7; 0.9) | 0.9 (0.8; 1.1) |
| Rich | 1.6 (0.9; 2.8) | 0.3 (0.03;2.2) | 0.6** (0.5; 0.9) | 0.7**(0.6; 0.9) | 0.9 (0.7; 1.1) |
| **Sources of drinking water** |  |  |  |  |  |
| Piped/Public tap/stan | 1 | 1 | 1 | 1 | 1 |
| Tube well or borehole | 0.9 (0.7; 1.2) | 1.0 (0.7; 1.5) | 1.2 (0.9; 1.6) | 0.8 (0.7; 1.0) | 0.8 (0.7; 1.0) |
| Well | 0.9 (0.6; 1.2) | 0.9 (0.6; 1.4) | 0.9 (0.7; 1.3) | 0.8* (0.6; 0.9) | 0.9 (0.8; 1.1) |
| Spring/river/lake/other | 0.8 (0.5; 1.3) | 1.1 (0.8; 1.7) | 1.0 (0.7; 1.4) | 0.7**(0.6;0.9) | 1.0 (0.8; 1.2) |
| ***Child level variables*** |  |  |  |  |  |
| **Sex of the child** |  |  |  |  |  |
| Female | 1 | 1 | 1 | 1 | 1 |
| Male | 1.2**(1.1; 1.4) | 0.9 (0.8; 1.2) | 1.2* (1.04; 1.4) | 1.2***(1.1; 1.3) | 1.0 (0.9; 1.1) |
| **Age in months** |  |  |  |  |  |
| 0-6 | 1 | 1 | 1 | 1 | 1 |
| 07-12 | 2.9***(2.3; 3.9) | 2.7***(1.9; 3.8) | 2.2***(1.7; 3.01) | 2.6***(2.2; 3.1) | 3.2***(2.7; 3.8) |
| 13-18 | 3.1***(2.4; 4.1) | 2.4***(1.7; 3.4) | 2.7***(1.9; 3.6) | 2.7***(2.3; 3.2) | 3.0 (2.6; 3.6) |
| 19-24 | 2.4***(1.8; 3.2) | 2.5*** (1.7; 3.7) | 2.9***(2.1; 4.0) | 2.1***(1.7; 2.5) | 2.6***(2.2; 3.1) |
| 25-30 | 1.8***(1.3; 2.4) | 1.6*(1.1: 2.4) | 1.6**(1.2;2.2) | 1.6***(1.4; 2.0) | 1.8*** (1.5; 2.2) |
| 31-36 | 1.5* (1.1; 2.1) | 1.8* (1.1; 2.8) | 1.4 (0.9; 1.9) | 1.1 (0.9; 1.4) | 1.6*** (1.3; 1.9) |
| 37-42 | 0.8 (0.6; 1.1) | - | 0.9 (0.7; 1.4) | 1.1 (0.9; 1.4) | 1.1 (0.9; 1.4) |
| 42-48 | 0.7 (0.5; 1.1) | - | 0.6* (0.4; 0.9) | 0.9 (0.7; 1.1) | 0.9 (0.8; 1.2) |
| 48-59 | 0.6**(0.5; 0.9) | - | 0.4***(0.3; 0.6)4 | 0.7**(0.6; 0.9) | 0.7 (0.6; 0.9) |
| **Has Measles vaccine** |  |  |  |  |  |
| No | 0.8(0.7; 1.0) | 0.9 (0.8; 1.3) | 0.7 (0.6; 0.9) | 0.9 (0.8; 1.0) | 0.8 (0.8; 0.9) |
| Yes | 1 | 1 | 1 | 1 | 1 |
| **Size of child at birth** |  |  |  |  |  |
| Large | 1 | 1 | 1 | 1 | 1 |
| Average | 1.2 (0.9; 1.4) | 1.2 (0.9; 1.6) | 1.2 (0.9; 1.4) | 0.8* (0.8; 0.9) | 0.8***(0.7; 0.9) |
| Small | 1.4**(1.1; 1.7) | 1.2 (0.8; 1.7) | 1.4**(1.1; 1.8) | 1.2**(1.1; 1.4) | 1.1 (0.9; 1.2) |
| DK (Don't know) | 1.8 (0.8; 4.2) | 1.5(0.6; 3.5) | 1.8 (0.7; 4.5) | 0.6 (0.4; 0.1) | 0.4 (0.3; 1.0) |
|  |  |  |  |  |  |
| ***Constant*** | 0.5*** (0.03; 0.10) | 0.08***(0.04;0.2) | 0.09***(0.05; 0.18) | 0.07*** (0.05; 0.11) | 0.06*** (0.04; 0.1) |
| ***Community Level SD*** | 0.71 (0.60; 0.84) | 0.63 (0.47; 0.84) | 0.77 (0.6; 0.9) | 0.82 (0.75; 0.91) | 0.87 (0.80; 0.95) |
| ***ICC*** | 0.13 (0.10; 0.17) | 0.11 (0.06; 0.18) | 0.15 (0.11; 0.21) | 0.17 (0.14; 0.20) | 0.19 (0.16; 0.21) |

Appendix 2: Multilevel Logistic Regression (Count…)

|  | **Niger** | | | |
| --- | --- | --- | --- | --- |
|  | **1992** | **1998** | **2006** | **2012** |
| ***Variable*** | **AOR (95% CI)** | **AOR (95% CI)** | **AOR (95% CI)** | **AOR (95% CI)** |
| ***n*** |  |  |  |  |
| **Household size** |  |  |  |  |
| 1-3 | 1 | 1 | 1 | 1 |
| 4-6 | 0.8 (0.62; 1.1) | 0.9 (0.7; 1.2) | 0.9 (0.7; 1.2) | 08 * (0.6; 0.9) |
| 7&+ | 0.8 (0.6; 1.1) | 0.9 (0.8: 1.3) | 0.9 (0.7; 1.2) | 0.7(0.6; 0.9) |
| **Sex of the head of household** |  |  |  |  |
| Male | 1 | 1 | 1 | 1 |
| Female | 1.4*(1.03; 1.8) | 0.9(0.7; 1.2) | 1.0 (0.9; 1.2) | 0.9 (0.7; 1.1) |
| **Age of the mother** |  |  |  |  |
| 15-19 | 1 | 1 | 1 | 1 |
| 20-24 | 0.9 (0.7; 1.2) | 1.1 (0.9; 1.4) | 1.0 (0.8; 1.3) | 0.9 (0.7; 1.1) |
| 25-29 | 0.9 (0.8; 1.3) | 1.1(0.8;1.4) | 1.0(0.8; 1.2) | 0.9 (0.7; 1.1) |
| 30-34 | 1.0 (0.7; 1.3) | 1.1(0.9; 1.4) | 0.9 (0.7; 1.1) | 0.9 (0.7; 1.2) |
| 35-39 | 1.1 (0.8; 1.5) | 1.1 (0.9; 1.5) | 0.8 (0.6; 1.1) | 1.0 (0.8; 1.3) |
| 40-44 | 1.1 (0.8; 1.6) | 1.3 (0.9; 1.8) | 0.1(0.8; 1.5) | 0.9 (0.7; 1.3) |
| 45-49 | 1.0 (0.6; 1.8) | 1.6 (0.9; 2.9) | 1.1 (0.7; 1.7) | 0.9 (0.6; 1.5) |
| **Place of residence** |  |  |  |  |
| Urban | 1 | 1 | 1 | 1 |
| Rural | 1.4*(1.0; 1.9) | 1.1 (0.8; 1.5) | 1.0 (0.7; 1.4) | 0.9 (0.7; 1.3) |
| **Mother Occupation** |  |  |  |  |
| Not working | 1 | 1 | 1 | 1 |
| Sale | 1.1(0.9; 1.3) | 1.3**(1.1; 1.5) | 1.3**(1.1; 1.5) | 1.1 (0.9; 1.3) |
| Agriculter | 1.4**(1.1; 1.7) | 1.1 (0.9; 1.4) | 1.4***(1.2; 1.7) | 1.5**(1.1; 2.1) |
| Manual | 1.4**(1.1; 1.8) | 1.0 (0.8; 1.2) | 1.2 (0.9; 1.5) | 1.0 (0.8; 1.3) |
| **Mother's education level** |  |  |  |  |
| No Education | 1 | 1 | 1 | 1 |
| 1-6 years | 0.9 (0.7; 1.2) | 1.1 (0.8; 1.3) | 0.9 (0.7; 1.1) | 1.1 (0.9; 1.3) |
| 7&+ years | 0.5** (0.4; 0.8) | 0.8 (0.5; 1.2) | 0.6* (0.4; 1.5) | 1.0 (0.8; 1.3) |
| **Has Electricity** |  |  |  |  |
| No | 1.2 (0.8; 1.7) | 1.0 (0.7; 1.5) | 1.2 (0.9; 1.6) | 1.1 (0.9; 1.5) |
| Yes | 1 | 1 | 1 | 1 |
| **Has Television** |  |  |  |  |
| No | 0.9 (0.6; 1.3) | 1.2 (0.8; 1.9) | 1.0 (0.7; 1.4) | 0.9 (0.7; 1.3) |
| Yes | 1 | 1 | 1 | 1 |
| **Has Refrigerator** |  |  |  |  |
| No | 1.1 (0.7; 1.8) | 0.8 (0.5; 1.4) | 0.8 (0.5; 1.2) | 0.8(0.6; 1.1) |
| Yes | 1 | 1 | 1 | 1 |
| **Has Bicycle** |  |  |  |  |
| No | 0.9 (0.7; 1.3) | 1.2 (0.9; 1.5) | 0.8 (0.7; 1.0) | 0.9 (0.8; 1.2) |
| Yes | 1 | 1 | 1 | 1 |
| **Has Motorcycle** |  |  |  |  |
| No | 1.03 (0.7; 1.4) | 0.8 (0.5; 1.1) | 0.9 (0.8; 1.2) | 1.0 (0.9; 1.2) |
| Yes | 1 | 1 | 1 | 1 |
| ***Immediat environment and hygiene*** |  |  |  |  |
| Type of toilette |  |  |  |  |
| Flush/upgraded latrin | 1 | 1 | 1 | 1 |
| Traditional latrines | 1.3 (0.7; 2.4) | 0.8 (0.4; 1.8) | 1.2 (0.7; 2.0) | 1.3 (0.9; 1.8) |
| Lack of toilet | 1.5(0.8; 2.8) | 0.9 (0.4; 1.9) | 1.2 (0.7; 2.2) | 1.3(0.9; 1.9) |
| Other type | 2.2 (0.9; 5.9) | 2.5 (0.7; 9.4) | 2.0 (0.8; 4.9) | 1.7 (0.8; 3.4) |
| **Quality of Main floor material** |  |  |  |  |
| Poor | 1 | 1 | 1 | 1 |
| Middle | 1.0 (0.8; 1.3) | 0.7* (0.5; 0.9) | 0.8 (0.7; 2.0) | 0.9 (0.7; 1.2) |
| Rich | 1.1 (0.5; 2.6) | 0.6 (0.2; 1.5) | 0.7 (0.3; 1.4) | 1.4**(1.1; 3.4) |
| **Sources of drinking water** |  |  |  |  |
| Piped/Public tap/stan | 1 | 1 | 1 | 1 |
| Tube well or borehole | 1.0 (0.8; 1.4) | 0.9 (0.7; 1.3) | - | 0.9 (0.8; 1.2) |
| Well | 0.9 (0.5; 1.4) | 0.8 (0.5; 1.4) | 1.1 (0.8; 1.4) | 0.8 (0.7; 1.0) |
| Spring/river/lake/other | 0.7 (0.6; 1.0) | 1.1 (0.8; 1.5) | 0.7 (0.4;1.2) | 1.3 (0.9; 1.9) |
| ***Child level variables*** |  |  |  |  |
| **Sex of the child** |  |  |  |  |
| Female | 1 | 1 | 1 | 1 |
| Male | 1.1 (0.9; 1.2) | 1.1(0.9; 1.2) | 1.0 (0.9; 1.1) | 1.1(0.9; 1.2) |
| **Age in months** |  |  |  |  |
| 0-6 | 1 | 1 | 1 | 1 |
| 07-12 | 2.6***(2.1; 3.3) | 2.3***(1.9; 2.8) | 2.4*** (1.9; 2.9) | 2.4***(1.8; 2.9) |
| 13-18 | 1.9***(1.5; 2.5) | 1.4**(1.1; 1.8) | 2.4***(1.9;2.9) | 1.7***(1.3; 2.1) |
| 19-24 | 1.9***(1.4; 2.4) | 1.3* (1.1; 1.7) | 1.9***(1.5; 2.4) | 1.2(0.9; 1.5) |
| 25-30 | 1.1 (0.9; 1.4) | 1.1 (0.9; 1.4) | 1.4**(1.1; 1.8) | 1.1(0.8; 1.3) |
| 31-36 | 0.8 (0.6; 1.02) | 0.8 (0.6; 1.1) | 1.1(0.9; 1.4) | 0.6 (0.5; 0.8) |
| 37-42 | 0.5***(0.4; 0.7) | - | 0.7 (0.5; 0.9) | 0.5***(0.3; 0.6) |
| 42-48 | 0.5***(0.3; 0.6) | - | 0.7 **(0.5; 0.9) | 0.4***(0.3; 0.5) |
| 48-59 | 0.4***(0.3; 0.5) | - | 0.5***(0.4; 0.6) | 0.2***(0.2; 0.3) |
| **Has Measles vaccine** |  |  |  |  |
| No | 1.0 (0.9; 1.2) | 1.0 (0.8: 1.2) | 0.8 (0.7; 0.9) | 1.0 (0.9; 1.2) |
| Yes | 1 | 1 | 1 | 1 |
| **Size of child at birth** |  |  |  |  |
| Large | 1 | 1 | 1 | 1 |
| Average | 1.0 (0.9; 1.2) | 0.9 (0.8; 1.1) | 0.8* (0.7; 0.9) | 0.9 (0.8; 1.1) |
| Small | 1.4***(1.2; 1.7) | 1.02 (0.8; 1.2) | 1.1 (0.9; 1.3) | 1.2*(0.1; 1.5) |
| DK (Don't know) | 1.3 (0.6; 3.2) | 0.4 (0.1; 2.2) | 0.9 (0.5; 1.7) | 0.6 (0.4; 1.0) |
|  |  |  |  |  |
| ***Constant*** | 0.16***(0.07; 0.3) | 0.4***(0.2; 1.1) | 0.2***(0.1; 0.4) | 0.16***(0.10; 0.27) |
| ***Community Level SD*** | 0.50(0.40; 0.60) | 0.43 (0.35; 0.56) | 0.60 (0.51; 0.70) | 0.65 (0.56; 0.75) |
| ***ICC*** | 0.07 (0.05; 0.10) | 0.06 (0.04; 0.09) | 0.10 (0.07; 0.13) | 0.11(0.09; 0.15) |
